# HpeNet: Co-expression Network Database for *de novo* Transcriptome Assembly of *Paeonia lactiflora* Pall

**DOI:** 10.3389/fgene.2020.570138

**Published:** 2020-10-21

**Authors:** Minghao Sheng, Jiajie She, Wenying Xu, Yan Hong, Zhen Su, Xiaodong Zhang

**Affiliations:** ^1^Beijing Agricultural Biotechnology Research Center, Beijing Engineering Research Center of Functional Floriculture, Beijing Academy of Agriculture and Forestry Science, Beijing, China; ^2^State Key Laboratory of Plant Physiology and Biochemistry, College of Biological Sciences, China Agricultural University, Beijing, China

**Keywords:** *Paeonia lactiflora* Pall., *de novo* assembly, functional annotation, co-expression network, database

## Abstract

The herbaceous peony (*Paeonia lactiflora* Pall.) is a well-known ornamental flowering and pharmaceutical plant found in China. Its high medicinal value has long been recognized by traditional Chinese medicine (as *Radix paeoniae Alba* and *Radix paeoniae Rubra*), and it has become economically valued for its oilseed in recent years; like other *Paeonia* species, it has been identified as a novel resource for the α-linolenic acid used in seed oil production. However, its genome has not yet been sequenced, and little transcriptome data on *Paeonia lactiflora* are available. To obtain a comprehensive transcriptome for *Paeonia lactiflora*, RNAs from 10 tissues of the *Paeonia lactiflora* Pall. cv Shaoyou17C were used for *de novo* assembly, and 416,062 unigenes were obtained. Using a homology search, it was found that 236,222 (approximately 57%) unigenes had at least one BLAST hit in one or more public data resources. The construction of co-expression networks is a feasible means for improving unigene annotation. Using in-house transcriptome data, we obtained a co-expression network covering 95.13% of the unigenes. Then we integrated co-expression network analyses and lipid-related pathway genes to study lipid metabolism in *Paeonia lactiflora* cultivars. Finally, we constructed the online database HpeNet (http://bioinformatics.cau.edu.cn/HpeNet) to integrate transcriptome data, gene information, the co-expression network, and so forth. The database can also be searched for gene details, gene functions, orthologous matches, and other data. Our online database may help the research community identify functional genes and perform research on *Paeonia lactiflora* more conveniently. We hope that *de novo* transcriptome assembly, combined with co-expression networks, can provide a feasible means to predict the gene function of species that do not have a reference genome.

## Introduction

*Paeonia lactiflora* Pall., commonly known as the herbaceous peony, is an ornamental flowering plant, well-known around the world. It is cultivated in more than 50 countries, including China, United States, New Zealand, and Turkey (Zhao et al., 2016). It has significant medicinal value (Yang et al., 2020) and can be used together with liquorice to treat a range of conditions, including spasmolysis; it also provides pain relief and calm (Wang et al., 2009). In recent years, the oil of *Paeonia lactiflora* has attracted increasing attention because it contains a high proportion of polyunsaturated fatty acids (PUFAs). Using PUFAs in place of saturated fat can help reduce the risk for cardiovascular disease and coronary heart disease in humans (Mozaffarian et al., 2010; Rizos et al., 2012). *Paeonia lactiflora* has recently attracted greater research attention. However, unlike other model species, the genome of *Paeonia lactiflora* has not been published, and population genetic resources are lacking, inhibiting gene function investigation and molecular breeding at the whole-genome level.

Recent years have seen extraordinary progress in next-generation sequencing technologies and many discoveries in high-throughput technology. Eight flavonoid metabolic pathway genes have been isolated, and the expression patterns of these eight genes in the flowers have been investigated (Zhao et al., 2012), enabling the mechanism of *Paeonia lactiflora* flower diversity to be described. It has also been found via transcriptome data analyses that the inhibition of anthocyanin biosynthesis mediates yellow formation (the color of the inner-petal is yellow) in *Paeonia lactiflora* (Zhao et al., 2014). Zifengyu and Hongyanzhenghui have been identified as thermo-tolerant and moderately thermo-tolerant cultivars, respectively. Analyses of the transcriptome data of these two cultivars have led to the detection of 161 heat stress response genes (Hao et al., 2016). Some miRNAs that might be involved in heat stress response have also been identified by miRNA sequencing (Hao et al., 2017). In addition, a combination of transcriptome and isobaric tags for relative and absolute quantification proteome (iTRAQ) analyses have revealed that APETALA2 regulates the development of flower shape (Wu et al., 2018). However, unlike other well-studied species, little transcriptome data have been assembled for *Paeonia lactiflora*, and these data focus on specific tissues and different cultivars. It is difficult to study *Paeonia lactiflora* at the whole-genome level using only available transcriptome data. These data have been generated in response to scientific questions, so certain tissue-specific genes and regulated genes are unlikely to have been detected. Additionally, transcriptome data from different cultivars show large differences, which make it more difficult to compare transcriptome data.

Without a reference genome for *Paeonia lactiflora*, *de novo* transcriptome assembly, a proven technology, can allow us to identify complete and contiguous mRNA sequences from RNA-seq data (Guttman et al., 2010; Grabherr et al., 2011; Davidson and Oshlack, 2014). There are great demands for high-quality transcriptome data from different tissues of the same cultivar. In this way, it is possible to predict and annotate the genes of *Paeonia lactiflora*, and these results can support investigations of gene function and molecular breeding. Using sequence similarity, identified assembled unigenes have been annotated by known datasets. In addition, co-expression analyses are a helpful way to improve functional annotation (Eisen et al., 1998; Walker et al., 1999). Gene function is closely related to gene-expression profiles, which can be established through the acquisition of transcriptome data (Inoue and Horimoto, 2017) (Moretto et al., 2019). Through the integration of available transcriptome datasets, constructed gene co-expression networks can link genes to specialized metabolic pathways, which can identify important genes and infer their potential functions (Rhee and Mutwil, 2014; Wisecaver et al., 2017; Salleh et al., 2018; van Dam et al., 2018). Co-expression networks must be constructed with high-quality and more complete transcriptome data. Transcriptome data have already been accumulated through model plants, and some co-expression network databases have been constructed, such as for *Arabidopsis*, rice, maize and cotton (You et al., 2015; Lee and Lee, 2017; You et al., 2017; Ma et al., 2018; Obayashi et al., 2018; Tian et al., 2018). For instance, ATTED-II is a co-expression database for nine plant species, with microarray data from 16,033 samples and RNA-seq data from 2,120 samples only for *Arabidopsis*. The RiceFREND database includes a large collection of rice microarray data drawn from various tissues at different stages of growth. The creation of similar resources for *Paeonia lactiflora* would require high-quality transcriptome data from the same cultivar and from different tissues to construct co-expression networks. Once this is done, functional annotation on the whole-genome level can be performed.

To improve the annotation of *Paeonia lactiflora*, we employed high-throughput sequencing technology and sequenced the transcriptomes of 10 tissues drawn from different growth stages of *Paeonia lactiflora* Pall. cv Shaoyou17C. With *de novo* assembly and gene function prediction, we produced reference transcriptome datasets. In addition, to further annotate the unigenes of *Paeonia lactiflora* and study gene functions, we constructed a co-expression network and created an online database, HpeNet^1^. The database contains transcriptome data, gene information, the co-expression network, and so forth. We hope that the resulting database will be beneficial to researchers conducting future work on *Paeonia lactiflora* Pall.

## Materials and Methods

### Plant Materials, Growth Conditions, and RNA Isolation

*Paeonia lactiflora* Pall. cv Shaoyou17C (a cultivar with high oilseed yield and high oil content bred by the Beijing Academy of Agriculture and Forestry Science), cv Hangbaishao (*Radix paeoniae Alba*), and cv Chishao (*Radix paeoniae Rubra*) were obtained from Yucheng County (Henan Province, China) and cultivated in open fields at Beijing Academy of Agriculture and Forestry Science in Beijing, China. All of the plants were 5 years old and were normally flowered and fruited, using standard agronomic practices.

To identify and annotate the transcripts more comprehensively, 10 tissues of *Paeonia lactiflora* Pall. cv Shaoyou17C were harvested from three biological replicates: the leaf (LY), flower (FY), kernel (KnY), ovary (OvY), stigma (StY), androecium (AnY), fibrous root (FRY), pod (CpY), seed coat (HsY) and four growth stages of seed (SY). In addition, the leaf (LMS) of *Paeonia lactiflora* Pall. cv Chishao and the flower (FW) and the leaf (LW) of *Paeonia lactiflora* Pall. cv Hangbaishao were also harvested from three biological replicates to isolate the RNA. All fresh samples were homogenized in liquid nitrogen and stored at −80°C.

Total RNA was isolated from each sample using TRIZOL^®^ reagent (Invitrogen, Carlsbad, CA, United States), according to the manufacturer’s protocol. The RNA was quantified and qualified using the Qubit^®^ RNA Assay Kit in a Qubit^®^ 2.0 Fluorometer (Life Technologies, Carlsbad, CA, United States) to measure RNA concentrations. RNA integrity was assessed using the RNA Nano 6000 Assay Kit of the Agilent Bioanalyzer 2100 system (Agilent Technologies, Santa Clara, CA, United States). Finally, the total RNA of 40 samples from different tissues and cultivars were obtained.

### Library Preparation and Sequencing

All library constructions and Illumina paired-end sequencing were done with Novogene. The sequencing libraries were generated using the NEBNext^®^ Ultra^TM^ RNA Library Prep Kit for Illumina^®^ (NEB, United States), following the manufacturer’s recommendations. Poly-T oligo-attached magnetic beads were used to purify the mRNA from total RNA. The quality of these libraries was assessed on the Agilent Bioanalyzer 2100 system. The libraries were prepared from three biological replicates of 10 tissues, sequenced on the IlluminaHiSeq^TM^ 2000 platform, following standard protocols. The paired-end reads were generated in 150 bp format.

### Data Processing and Transcriptome *de novo* Assembly

The experimental design and mRNA-seq procedures are shown in Supplementary Figure 1. The Q20, Q30, GC-content, and sequence duplication levels of the clean data were calculated with FastQC. We defined the number of bases using a mass value of Q_phred_ ≤ 20, which accounted for more than 50% of the entire read as low-quality reads. To generate clean data, all reads containing adapter, reads containing Ploy-N of more than 10%, and low-quality reads were removed. All of the clean reads of *Paeonia lactiflora* Pall. cv Shaoyou17C were used for *de novo* transcriptome assembly using Trinity (r20140413p1) (Grabherr et al., 2011), and all of the parameters were set to the defaults (min_contig_length 200, min_glue 2, min_kmer_cov 2). The transcript sequence obtained by Trinity was used as a reference sequence for subsequent analyses. Then Corset (Davidson and Oshlack, 2014) was employed to create a hierarchical cluster from the transcripts according to the number of mapped reads and expression patterns. After clustering was done with the Corset hierarchy, the longest cluster sequence (unigene) was obtained to produce functional annotation and transcriptome expression analyses.

### Functional Annotation of Unigenes

We predicted the annotations of the unigenes in *Paeonia lactiflora* Pall. cv Shaoyou17C using sequence alignment searches for homologous proteins against seven authoritative public databases (Altschul et al., 1997), including NCBI non-redundant protein sequences (NR^2^), NCBI non-redundant nucleotide sequences (NT^2^), Protein family (Pfam (Finn et al., 2008)^3^), the euKaryotic Ortholog Groups (KOG^4^), Swiss-Prot^5^, Kyoto Encyclopedia of Genes and Genomes (KEGG^6^), and Gene Ontology (GO^7^). Blast2GO software (b2g4pipe_v2.5; Gotz et al., 2008) was used to predict the GO annotation of unigenes with an E-value cutoff of 1E–6. KEGG annotations were generated using the KEGG Automatic Annotation Server (KAAS, r140224; (Moriya et al., 2007), with an E-value cutoff of 1E–10. We obtained the domain architecture of the protein family from the Pfam database^8^ (Finn et al., 2010) and the HMMER 3.0 package, with the cutoff for the E-value set to 0.01. The NT database annotation was based on NCBI BLAST (v2.2.28+), and an E-value of 1E–5 was chosen as the cutoff. The remaining three databases, NR, Swiss-Prot, and KOG, were searched using diamond software (v0.8.22), with E-values of 1E–5, 1E–5, and 1E–3, respectively. To identify the possible orthologous genes in *Arabidopsis*, the unigenes were compared with The *Arabidopsis* Information Resource 10 (TAIR10) for *Arabidopsis thaliana*^9^ using the BLASTX method, with an E-value cutoff of 1E–5, and the top hits were selected as orthologous genes.

### Transcriptome Expression Analyses

The unigene expression level for each sample was estimated using RSEM (Li and Dewey, 2011). RSEM does not require a reference genome and, it is capable of handling multiple mapped reads. First, clean data were mapped back onto the assembled transcriptome. Then, read counts for each unigene were obtained from the mapping results, and the read counts were converted into fragments per million mapped reads (FPKM) to analyze the expression level of the unigene. We set FPKM > 0.3 as the minimum FPKM value to call a unigene as an expressed unigene. This threshold is recommended by mainstream magazines and, can also reflect gene expression levels well. To analyze the differential gene expression, the DESeq package (Love et al., 2014) was employed, and the methods of Benjamini and Hochberg were used to control the false discovery rate and adjust the resulting P-value. We screened the threshold for Padj < 0.05 and |log2FoldChange| > 1 as differentially expressed genes (DEGs).

### Co-expression Network Construction Algorithm

Pearson’s correlation coefficient (PCC) was used to calculate the correlations between the expression patterns of two unigenes among all of the samples, and mutual rank (MR) took a geometric average of the PCC rank (Obayashi and Kinoshita, 2009; Klinkenberg et al., 2014; Aoki et al., 2016). The mathematical formulas for PCC and MR are as follows:

(1) $$PCC=\frac{\sum_{i=1}^{n}\left(x_{i}-\bar{x}\right)\left(y_{i}-\bar{y}\right)}{\sqrt{\sum_{i=1}^{n}{\left(x_{i}-\bar{x}\right)}^{2}\cdot \sum_{i=1}^{n}{\left(y_{i}-\bar{y}\right)}^{2}}}$$

(2) $$MR\left(AB\right)=\sqrt{\left(Rank\left(A\to B\right)\times Rank\left(B\to A\right)\right)}$$

where *X* and *Y* represent the FPKM values for two genes, and *n* represents the number of samples; *i* represents samples in the formula under different conditions. To calculate the MR values, we ranked all gene pairs from high to low by their PCC values. Using formula (2), MR takes the geometric average of the relative positions of genes A and B based on the ranked PCC values (You et al., 2015, 2017; Ma et al., 2018; Tian et al., 2018; She et al., 2019).

### Statistical Test Method

Fisher’s exact test was used to compute significance in gene-set enrichment analyses (Yi et al., 2013). The mathematical formula for this method is given as follows:

(3) $$P=\frac{\left(\begin{array}{c} n\\ k \end{array}\right)\left(\begin{array}{c} N-n\\ K-k \end{array}\right)}{\left(\begin{array}{c} N\\ K \end{array}\right)}$$

where *N* represents the number of all protein-coding unigenes, *n* represents the gene number in the query gene list, *K* is the gene number for a certain background gene set, and *k* is the number of overlapping genes between the query gene list and the background gene set.

### Search and Visualization Platform

The database was constructed in the LAMP (Red Hat Linux + Apache^10^ + MySQL^11^ + PHP^12^) environment. The network visualization was dynamically displayed in Cytoscape.js^13^, an open-source java script package (Franz et al., 2016).

## Database Construction and Content

### Transcriptome of 10 *Paeonia lactiflora* Pall. Tissues Determined via Sequencing and *de novo* Assembly

*Paeonia lactiflora* Pall. cv Shaoyou17C is a hybrid cultivar of *Paeonia lactiflora* Pall. cv Chishao and *Paeonia lactiflora* Pall. cv Hangbaishao. *Paeonia lactiflora* Pall. cv Shaoyou17C has high seed production and oil content bred. By sequencing and analyzing its transcriptome, we wish to discover the related genes regulating the high oil content and high proportion of PUFAs. There is no reference genome for *Paeonia lactiflora* Pall at present, so we chose transcriptome sequencing with *de novo* assembly to investigate transcripts in the 10 tissues of *Paeonia lactiflora* Pall. cv Shaoyou17C. The total RNA of each sample with three biological replicates (except for the four stages of the seed) was individually isolated, and the transcriptome profiles were generated on the Illumina HiSeq2000 platform (see detailed description in section “MATERIALS AND METHODS”). After data cleaning and quality control, a total of 2 billion 327 million high-quality 150 bp paired–end reads, at about 349.05 Gb, were generated (Supplementary Table 1).

**FIGURE 1:**
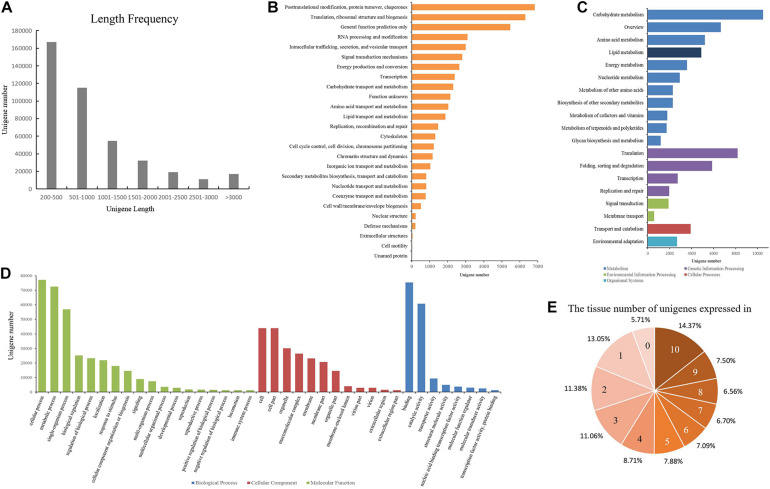
Data quality and annotation of unigenes assembled with mRNA-seq. **(A)** Length distribution of unigenes. **(B)** Unigenes annotated using the Eukaryotic Orthologous Groups (KOG) tool. All unigenes were divided into 26 groups. **(C)** Functional annotation of the unigenes based on the KEGG classification. The numbers of unigenes mapped to each pathway group are shown in the bar chart. Colors indicate KEGG categories: blue for metabolism, purple for genetic information processing, green for environmental information processing, red for cellular processes, and cyan for organismal systems. **(D)** Gene ontology (GO) classification of the unigenes. Each bar represents the number of unigenes mapped to each GO category. Bar color indicates GO category: green for biological process (BP), red for cellular component (CC), and blue for molecular function (MF). **(E)** Number of the tissues in which each unigene is expressed.

To obtain a high-quality and comprehensive transcriptome, all of the high-quality reads from 10 tissues of Shaoyou17C were used for transcript assembly. The overlapping information of the high-quality reads provided a total of 519,560 transcripts for successful assembly using Trinity software. Then Corset software was used to cluster contigs, count reads, and remove redundancy from the assembly. At the end of this process, we obtained 416,062 unigenes. Next, we assessed the quality of the assembled transcriptome. The lengths of these unigenes ranged from 201 bp to 13,684 bp, with a mean length of 965 bp and an N50 of 1,462 bp (Supplementary Table 2). The length frequency of assembled unigenes was further analyzed (Figure 1A). We found that 167,108 unigenes (40.16%) were less than 500 bp, and 114,982 unigenes (27.64%) ranged from 500 to 1000 bp. There were 117,052 unigenes (20.90%) that ranged from 1000 bp to 3000 bp and 16,920 unigenes (11.3%) that were longer than 3000 bp (Figure 1A).

### Functional Annotation of the Assembled Unigenes

All of the unigenes were annotated using the results of the BLAST comparison public databases, including the NR, NT, Swiss-Prot, Pfam, KOG, and the *Arabidopsis* protein (TAIR10) databases. The results showed that 197,488 (47.46%) and 129,014 (31%) unigenes were annotated with the NR (E-value ≤ 1E–5) and NT (E-value ≤ 1E–5), respectively. Next, we searched the predicted unigene sequences against two of the other databases. A homology search in the Pfam database (E-value ≤ 0.01) indicated that 139,121 transcripts were annotated at least once, while in Swiss-Prot database (E-value ≤ 1E–5), 138,800 unigenes were annotated. To provide further functional prediction and classification, all assembled unigenes were aligned using the KOG database (E-value ≤ 1E–3). A total of 45,348 unigenes were annotated into 26 clusters (Figure 1B). Putative functions were assigned for over 50% of the unigenes (Table 1).

**TABLE 1:** Annotation of unigenes based on multiple database resources.

Source	Number of unigenes	Percentage (%)	E-value
NR	197488	47.46%	1E–5
NT	129014	31%	1E–5
Pfam domains	139121	33.43%	0.01
Swiss-Prot	138800	33.36%	1E–5
KOG annotation	45348	10.89%	1E–3
GO annotation entries	142163	34.16%	1E–10
KEGG pathways	72433	17.40%	1E–6
Orthologous pairs in *Arabidopsis*	166486	40.01%	1E–5
Annotated in all databases	25291	6.07%	–
Annotated in at least one database	236222	56.77%	–
Total unigenes	416062	–	–

KEGG annotation provides information on the molecular network. A total of 72,433 (17.4%) unigenes with E-values < 1E–10 were mapped to 130 pathways belonging to all five major categories of KEGG (Figure 1C, Supplementary Table 3). Most unigenes were annotated in the metabolism category, with 43,031 unigenes. Lipid metabolism is a metabolic pathway related to oil content featuring 4880 unigenes. The oil of *Paeonia lactiflora* contains a high proportion of linolenic acid. Of all of the pathways, ko00061 (Fatty acid biosynthesis) and ko00071 (Fatty acid degradation) were most likely to significantly contribute to linolenic acid biosynthesis. These had 404 unigenes and 612 unigenes, respectively (Table 2).

**TABLE 2:** Unigenes related to lipid metabolism, based on KEGG classification.

KEGG Pathway	Gene Number
Biosynthesis of unsaturated fatty acids	481
Linoleic acid metabolism	161
Alpha-linolenic acid metabolism	652
Fatty acid biosynthesis	404
Fatty acid degradation	612
Fatty acid elongation	174
Glycerolipid metabolism	437
Glycerophospholipid metabolism	665
Sphingolipid metabolism	284
Steroid biosynthesis	333
Synthesis and degradation of ketone bodies	58
Arachidonic acid metabolism	115
Ether lipid metabolism	214
Cutin, suberine, and wax biosynthesis	290

GO is an international standardized functional gene classification that associates GO terms with other functional annotations of *de novo* assembly unigenes from homology searches. There were 142,163 unigenes found with at least one GO term, obtained using Blast2GO software (E-value ≤ 1E–6). The major categories in GO [biological process (BP), molecular function (MF), and cellular component (CC)] are represented in Figure 1D. Among the secondary entries, 292 unigenes, 25,177 unigenes and 72,426 unigenes were annotated as growth (GO:0040007), biological regulation (GO:0065007) and metabolic process (GO:0008152), respectively (Supplementary Table 4).

### Co-expression Network Construction

Approximately 56.77% of unigenes were annotated using public databases, (Table 1). To improve the functional annotation of the assembled unigenes and the functional genomics research for *Paeonia lactiflora* Pall. cv Shaoyou17C, we constructed a co-expression network. First, we checked the correlation of the replicates. RSEM was used to map the clean reads to the unigenes. In this mapping, about 69% to 85% of reads were mapped to the reference transcripts (Supplementary Table 5). From these mapping results, the FPKM value for each unigene in each sample was computed. Next, we calculated the correlation coefficient for each sample. Every three biological replicates in each sample had a high correlation (Supplementary Figure 2). The low correlation for the four seed stages could be due to the large differences between stages of seed growth. The expression patterns of the unigenes were checked, and then the distribution of expression for unigenes in different tissues was summarized. In all, 394,329 unigenes are expressed at least in one tissue and, 59,199 unigenes were expressed in all 10 tissues of Shaoyou17C (Figure 1E), and 28.43% were expressed in more than eight tissues. Half of the unigenes were detected in more than five tissues. These results indicate that most unigenes have good coverage and high quality.

Using transcriptome data, the PCC and MR (Umrania et al., 2017) algorithms were combined to construct a co-expression network. PCC and MR were computed to relate all pairs of unigenes based on a correlation algorithm (see detailed description in Methods). The PCC distributions (Supplementary Figure 3A) indicated that the co-expression network featured a similar normal distribution. To obtain the optimal co-expressed gene pairs, we set strict parameters. We found that the threshold of PCC had a cut-off of 0.7, and the MR top3 + MR ≤ 30, which were chosen to construct the co-expression network and ensured its coverage and creditability. There were 395,501 nodes in the constructed network, which covered 95.05% of unigenes in *Paeonia lactiflora* Pall. In all, there were 3,376,888 and 1,197,375 pairs in the positive co-expression network (PCC-value > 0) and negative co-expression network (PCC-value < 0), respectively. Whether it is in the positive or negative co-expression network, as the number of edges increases, the number of nodes decreases (Supplementary Figures 3B,C). The statistics for gene pairs suggest that positive and negative gene pairs were in accordance with the scale-free network.

### Tissue-Preferential Analyses and Comparative Analyses Between Different Cultivars

We investigated unigene-expression patterns in different tissues. For Shaoyou17C, the 10 aforementioned tissues (LY, FY, KnY, OvY, StY, AnY, FRY, CpY, HsY and SY) were subjected to co-expression network analyses. The gene expression value was overlaid onto the co-expression network to estimate whether a given gene was expressed in the given tissue.

Chishao has a low seed setting rate but large seeds, Hangbaishao has small seeds but a high seed setting rate, and Shaoyou17C combines the advantages of both. Further, because differences between different cultivars could be caused by DEGs, the expression patterns were compared between different cultivars. We obtained transcriptome data from the leaf tissue of three cultivars, namely, Shaoyou17C, Chishao, and Hangbaishao, and from the flower tissues of Shaoyou17C and Chishao. Each tissue had three independent biological replicates. Following previous studies, Padj < 0.05 and |log2FoldChange| > 1 were chosen as thresholds. DEGs were identified between two different cultivars in leaf and flower. Among the leaf samples, there were 46,736 DEGs, 52,850 DEGs, and 56,414 DEGs (Padj < 0.05 |log2FoldChange| > 1) between Shaoyou17C and Chishao, Shaoyou17C and Hangbaishao, and Chishao and Hangbaishao, respectively. In the flower of Shaoyou17C and Chishao, 25,541 unigenes were DEGs (Supplementary Table 6). We also checked the number of DEGs under a stricter threshold (Padj < 0.05 |log2FoldChange| > 2). For a more intuitive presentation, red and blue were chosen to represent up-regulated and down-regulated genes, respectively. All of the DEGs were overlaid onto a co-expression network (Supplementary Figure 4). Users are able to check the DEGs between different cultivars. We also provided a user-friendly interface to show the expression patterns of co-expressed unigenes in different tissues and cultivars.

### Construction of a Database With the Co-expression Network

**FIGURE 2:**
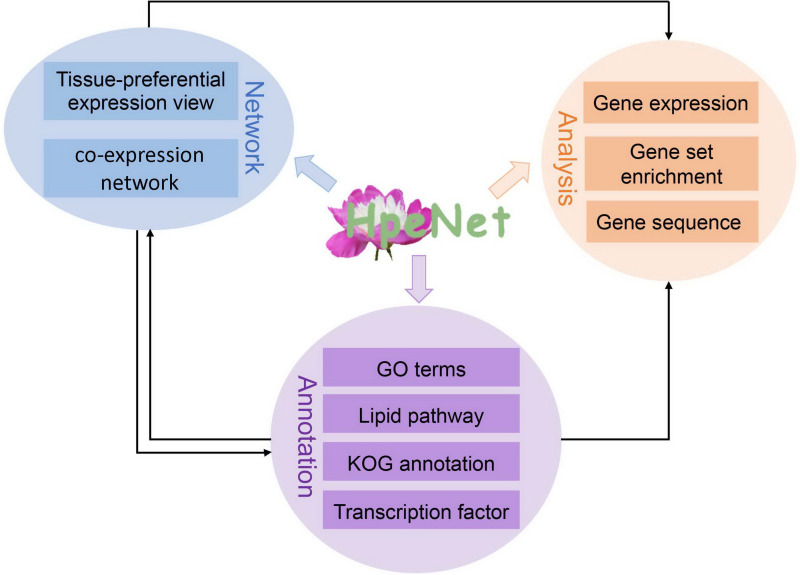
Database architecture. The HpeNet database is divided into three main sections: network, annotations, and analytical tools. Lines with different colors indicate different pieces.

Based on previous work, a database (HpeNet) was constructed for a co-expression network with functional analyses in *Paeonia lactiflora* Pall. This database was developed in the LAMP environment. It features three main parts: tissue-preferential expression view and co-expression network, including tissue-preferential and comparative analysis between different cultivars; functional annotation, including GO terms, the lipid pathway, KOG annotation, transcription factors, and Pfam domains, and so forth; and analysis tools for the co-expression network, including gene expression profiling analysis and gene set enrichment analysis (GSEA) (Figure 2). HpeNet can be accessed online at http://bioinformatics.cau.edu.cn/HpeNet/.

**FIGURE 3:**
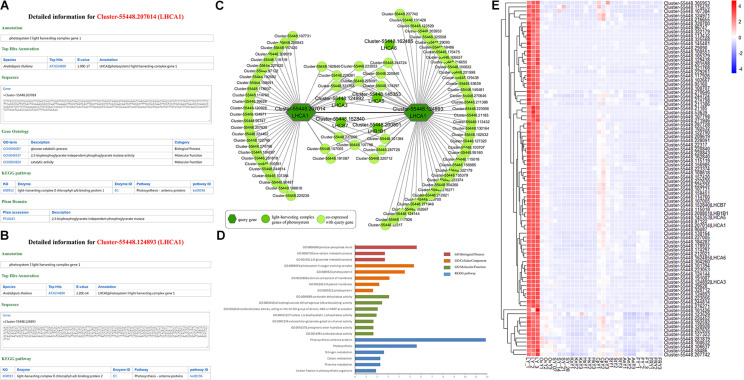
Comprehensive explorations for the function of LHCA1 (Cluster-55448.207014 and Cluster-55448.124893). **(A)** and **(B)** Individual gene detail pages for Cluster-55448.207014 and Cluster-55448.124893, respectively. **(C)** Co-expression network for the LHCA1 genes (Cluster-55448.207014 and Cluster-55448.124893) in *Paeonia lactiflora*. The other light-harvesting complex genes of photosystem are highlighted with a dark green circle. **(D)** GSEA enrichment analyses of all genes from the co-expression network for LHCA1. **(E)** Heatmap of all genes from the co-expression network expressed in different samples.

We took Cluster-55448.207014 as an example to illustrate the functions offered by HpeNet (Figure 3A). A gene detail search indicated that it was annotated as photosystem I light harvesting complex gene 1 (LHCA1). Then we explored possible functions of the unigene in the database. The annotation information found on the gene detail page shows that AT3G54890 (LHCA1) is the orthologous gene in *Arabidopsis thaliana*, and the sequence of Cluster-55448.207014 was presented in the database. GO annotation and the KEGG pathway were also available. This was annotated as “glucose catabolic process” (GO:0006007), “2,3-bisphosphoglycerate-independent phosphoglycerate mutase activity” (GO:0046537), “catalytic activity” (GO:0003824), and light-harvesting complex II chlorophyll a/b binding protein 1 (K08912). This suggests that Cluster-55448.207014 is involved in the glucose catabolic process and functions as a light-harvesting complex II a/b binding protein. The annotation information on the Pfam domain indicates that Cluster-55448.207014 has a PF10143 domain, which functions as 2,3-bisphosphoglycerate-independent phosphoglycerate mutase. Through a network search, users can obtain co-expressed relationships and detailed information on network members to enable further analyses, including network analysis (tissue-preferential analysis and comparative analysis between different cultivars), gene expression profiling analysis and gene set enrichment analysis. Using the lipid-related pathway, users can search a pathway of interest and find detailed information on key enzymes and conduct network analyses. With the supported analytical tools, users can conduct BLAST alignment and gene set enrichment analysis for the gene list of interest. Furthermore, a database manual is provided.

## Case Study

### Gene Network Analyses of Photosynthesis-Related Genes

Photosynthesis is the major source of energy for plant growth and development. In the higher plants, Photosystem I (PSI) and Photosystem II (PSII) harvest light in the thylakoid membrane. The peripheral light-harvesting complex of PSI (LHCI) is one of the two most important contents in PSI. It is composed of four LHCA complexes and is assembled into two heterodimers, LHCA1/4 and LHCA2/3. The study of the light-harvesting complex (LHC) genes of PSI and PSII in *Paeonia lactiflora* can help us understand its growth and development. Using orthologous genes, 175 LHC unigenes in *Paeonia lactiflora* were identified with 16 LHC unigenes in *Arabidopsis* (Supplementary Table 7). Cluster-55448.207014 and Cluster-55448.124893, both homologous to AT3G45890 (LHCA1), were selected, and their detailed information and co-expression networks were searched in the database (MR ≤ 50) (Figures 3A,B). In the KEGG pathway annotation, both of the unigenes were annotated with the light-harvesting complex KEGG term.

A co-expression relationship was found between Cluster-55448.207014 and Cluster-55448.124893 and the five other unigenes of the light harvesting complex, Cluster-55448.124892 (orthologous gene of LHCA3), Cluster-55448.145353 (orthologous gene of LHCA5), Cluster-55448.162485 (orthologous gene of LHCA6), Cluster-55448.152840 (orthologous gene of LHCB7), and Cluster-55448.200801 (orthologous gene of LHB1B1) were in the co-expression network (Figure 3C). To validate the networks and further investigate the function of the co-expressed genes, gene set enrichment analysis was conducted. The GO terms “photosystem I” (GO:0009522, FDR = 0.0237), “photosystem II oxygen evolving complex” (GO:0009654, FDR = 4.45E-06) and oxidoreductase activity, acting on the CH-OH group of donors, NAD or NADP as acceptor” (GO:0016616, FDR = 8.75E-03) were significantly enriched. Additionally, the co-expression genes were also associated with the KEGG terms “Photosynthesis-antenna proteins” (FDR = 1.33E-12) and “Carbon fixation in photosynthetic organisms” (FDR = 0.0124) (Figure 3D). Consistent with the findings for *Arabidopsis* and other species, Cluster-55448.207014, Cluster-55448.124893, and their co-expressed genes were closely related to photosynthesis. Next, the expression patterns for Cluster-55448.207014, Cluster-55448.124893, and their co-expressed genes were checked (Figure 3E). As with LHCA1 in *Arabidopsis*, the unigenes were preferentially expressed in the leaf and hardly expressed at all in other tissues, particularly the fibrous root. This corresponds to the organization of photosynthesis. Some unannotated unigenes in the network may also be involved in photosynthesis.

### Network Analyses for Genes Related to Oleate Desaturase

The seed oils were obtained through fatty acid biosynthesis, which includes synthesis, transport, and desaturation. The endoplasmic reticulum, plastids and cytosol were the main locations of reaction. (Bourgis et al., 2011; Li-Beisson et al., 2013). There are three types of enzymes in plant seeds that are primarily responsible for fatty acid composition: stearoyl-ACP desaturase, SAD (Klinkenberg et al., 2014), oleate desaturase, FAD2 (Okuley et al., 1994) and linoleate desaturase, FAD3 (Browse et al., 1993). They act in sequence to desaturate stearic acid (18:0) to oleic acid (18:1), to linoleic acid (18:2), and then to linolenic acid (18:3) (Miquel and Browse, 1992; Browse et al., 1993). In soybean, the largest oilseed crop, double mutants of the FAD2 and FAD3 genes result in high 18:1 and low 18:2 (Pham et al., 2012). Soybean contains 23% oleic acid, 54% linoleic acid, and only 8% linolenic acid (Wilson, 2004). Unlike that of oil-producing crops, the oil of *Paeonia lactiflora* Pall. cv Shaoyou17C contains a high proportion of PUFA. It has 35.28% oleic acid (18:1), 26.9% linoleic (18:2) acid, and 32.2% linolenic acid (18:3) content. This stimulated our great interest in investigating the differences between *Paeonia lactiflora* and other oilseed crops.

To investigate why Shaoyou17C seeds contain such a high proportion of linoleic acid and linolenic acid, the fatty acid synthesis pathway enzyme in Shaoyou17C was identified first through a prediction of the homologous genes in *Arabidopsis*. After genes with poor reproducibility were removed, we chose the median values of three repeats to represent the expression pattern for each tissue. A comparison of the expression pattern of fatty acid synthesis pathway genes in different tissues showed that an oleate desaturase, Cluster-55448.309693 (orthologous gene of FAD2), preferentially expressed in seed organ (Supplementary Table 8).

**FIGURE 4:**
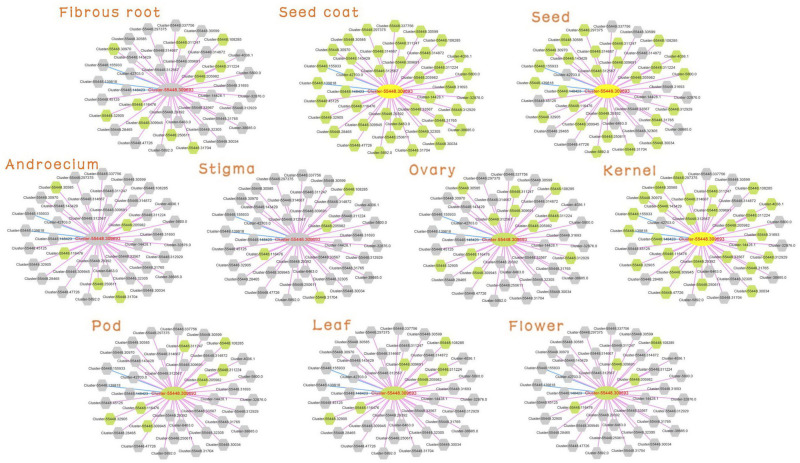
Expression view of FAD2 network in different tissues. Query gene and co-expressed genes highlighted with yellow and green circles, respectively. Gray circles represent genes without an expression profile in that tissue.

Detailed information on Cluster-55448.309693 was searched (MR ≤ 30) in the database. The tissue preferential network with expression of the unigenes in the 10 tissues is shown in Figure 4. The gray node was not expressed in the corresponding tissue. Consistent with the expression characteristic of Cluster-55448.309693, the network in seed, seed coat and kernel had more expressed genes, and the network structure was relatively complete. Cluster-55448.309693 together with its co-expressed genes did not express in fibrous root, androecium, stigma.

**FIGURE 5:**
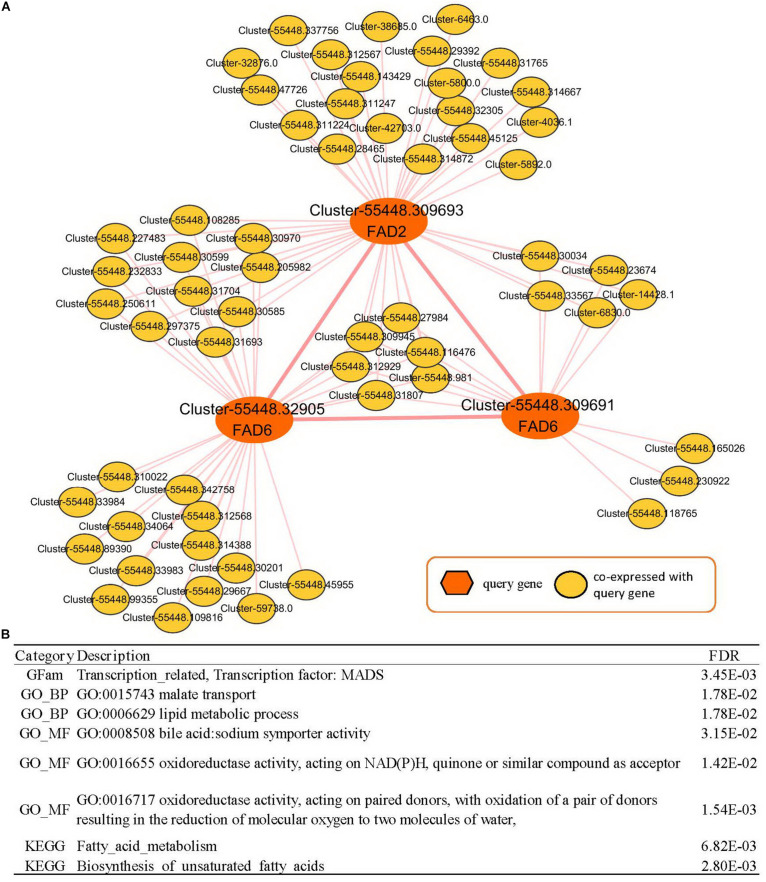
Co-expression network analyses of *Paeonia lactiflora* fatty acid desaturase genes. **(A)** Co-expression network of FAD2 (Cluster-55448.309693) and FAD6 (Cluster-55448.32905 and Cluster-55448.309691). **(B)** GSEA enrichment analyses for all genes from **(A)**. The results shown list the category, description, and FDR value.

The co-expressed genes of Cluster-55448.309693 contained two fatty acid desaturases, Cluster-55448.32905 and Cluster-55448.309691 (orthologous gene of FAD6), co-expressed with FAD2. Then the expression network of these three fatty acid desaturases was analyzed (Supplementary Table 9, Figure 5). In GSEA analyses, it was found that genes in the co-expression network were enriched with gene sets related to FA metabolism, such as the “lipid metabolic process” (GO:0006629, FDR = 0.0178), “oxidoreductase activity, acting on NAD(P)H, quinone or similar compound as acceptor” (GO:0016655, FDR = 0.0142), “Fatty acid metabolism” (FDR = 6.82E-03) and “biosynthesis of unsaturated fatty acids” (FDR = 2.80E-03). These could help us study seed yield and the oil content of peony. Therefore, in addition to FAD2, FAD6 might play an important role in fatty acid desaturation in *Paeonia lactiflora* seeds.

In summary, the transcriptome of peony was *de novo* assembled, with annotated and improved information on unigenes through the co-expression network. We developed an online database, HpeNet, that integrated transcriptome data, gene information, the co-expression network, and so forth. This made up for the lack of transcriptome data for *Paeonia lactiflora*. In addition, the database provided gene detail search, co-expressed network, lipid-related pathway gene inquiry, and several functional analytic tools that can help investigate gene function and molecular breeding.

## Discussion

We sequenced the transcriptomes of 10 tissues from different growth stages of *Paeonia lactiflora* Pall. cv Shaoyou17C. After the data were cleaned and quality control was implemented, a total of 2 billion 327 million reads with about 349.05 Gb transcriptome data were obtained. Then we *de novo* assembled the transcriptome of *Paeonia lactiflora* Pall. and as a result 416,062 unigenes were predicted. Furthermore, using public authoritative databases, 236,222 (approximately 57%) unigenes were annotated. In addition, a co-expression network was constructed to gather all available data into the online database HpeNet, which is easy for international users to access. The database also allows researcher to search by gene detail, gene function, and orthologous matches, as well as tools for co-expressed network analysis, lipid-related pathway gene inquiry, and several for functional analysis. We hope that our study will be beneficial to studies on *Paeonia lactiflora* Pall. and some related species in Ranales.

The genome of *Paeonia lactiflora* Pall. has not yet been decoded, and gene annotation is far from complete. The prohibitive costs associated with sequencing and assembling the genome make it infeasible to consider whole-genome sequencing in the near future as NGS technology evolves (Goodwin et al., 2016). Transcriptome analysis is an important way in which gene discovery can go forward, and the transcribed sequences contain a high content of functional information. To build a comprehensive transcriptome, we chose 10 representative tissues with three biological replicates at different stages of *Paeonia lactiflora* Pall. cv Shaoyou17C. To the best of our knowledge, this is the most comprehensive measurement of the *Paeonia lactiflora* transcriptome, and it covers various tissues of *Paeonia lactiflora*. High-quality sequences of unigenes were obtained through *de novo* assembly. This approach goes beyond supporting research on the molecular biology of *Paeonia lactiflora*, including gene cloning and homologous and phylogenetic analysis, and maybe beneficial to decoding its entire genome.

Co-expression network is an optimal instrument to use in exploring gene function. Many co-expression network databases have been constructed for model plants and important crops, such as ATTED-II (Obayashi et al., 2018), PlaNet (Proost and Mutwil, 2017), AraNet (Lee and Lee, 2017), RiceNet (Lee et al., 2015), RiceFREND (Sato et al., 2013), ccNET (You et al., 2017), MCENet (Tian et al., 2018) and WheatNet (Lee et al., 2017), but there are few co-expression databases for no-reference genome plants such as *Paeonia lactiflora* Pall. Annotation could help develop analyses of the function of unigenes and molecular breeding for *Paeonia lactiflora*.

Using the co-expression network, we found that LHCA1, Cluster-55448.207014, and Cluster-55448.124893 were co-expressed with five other genes of the light-harvesting complex, namely, LHCA3, LHCA5, LHCA6, LHCB7 and LHB1B1. In addition, Cluster-55448.309693 (orthologous gene of FAD2), an important fatty acid desaturase, was co-expressed with two other fatty acid desaturases, Cluster-55448.32905 and Cluster-55448.309691 (orthologous gene of FAD6). These results show that the network is reliable. Taking Cluster-55448.309693 as an example, comparative analyses between different cultivars indicated that Cluster-55448.309693 had a similar network structure in different cultivars, and fewer than three DEGs existed in the network. However, as we are limited by the data, only a few DEGs were noted in leaves and flowers. It is noteworthy that the network also contains a large number of unannotated unigenes. Using the database, it is possible to query the expression changes in co-expressed genes of different cultivars.

To facilitate the development of this information, we constructed HpeNet, a database with unigene information, a co-expression network, and analytical tools. We have not yet found any databases related to *Paeonia lactiflora* or even to any Ranales species. HpeNet may be the first online database for a Ranales species that offers gene information and co-expression networks. Using this database, users can query unigene information, including predicted annotation information, sequence information, and expression information for different tissues. We annotated 236,222 unigenes using seven public and authoritative databases. It is worth noting that about 57% of unigenes were annotated from public authoritative databases, a rate that is similar to that of many other *de novo* assembly plant transcriptomes, such as *Cuscuta pentagona* (Ranjan et al., 2014), *Lens culinaris* Medik. (Verma et al., 2013), and *Arceuthobium sichuanense* (Wang et al., 2016). This indicates that unigene annotation should be improved.

This study had limitations and it could be improved. Although 10 organs from the series growth stages of *Paeonia lactiflora* Pall. were included, some were excluded. This necessarily prevented us from being able to predict all genes. *De novo* assembly circumvents the need for a reference genome, but this approach has some deficiencies. Investigations have found that *de novo* assembly often generates fragments of a transcript or multiple contigs (Davidson and Oshlack, 2014). Furthermore, we obtained a large number of unigenes, although we employed Corset to merge the results and hierarchically cluster the contigs. Additionally, genes that share sequences, sequencing errors, variations and other issues can increase the number of unigenes (Vijay et al., 2013). Transcriptome data on seeds at different time points of development and on different tissues from flowers and seeds can be used for more detailed analyses. The reasons for the high proportion of unsaturated fatty acids, the key genes that regulate flowering, and the important genes that control the development of oil seed and other key functions. all require additional research.

In summary, through the transcriptomes of 10 tissues at different growth stages of *Paeonia lactiflora* Pall. cv Shaoyou17C, we obtained a comprehensive transcriptome with 416,062 unigenes. In addition, key insights were obtained from them through transcriptome profiling. Utilizing both sequence homology and co-expression networks, the functional annotation of the unigenes was refined and the functional genomic understanding of *Paeonia lactiflora* Pall. was developed. Further, HpeNet provides an online platform with transcriptome data, gene information, co-expression networks, and analytical tools. It will be convenient for researchers to obtain this information. This study improves our understanding of *Paeonia lactiflora* Pall. and provides a feasible way to study species without a reference genome.

## Data Availability Statement

The RNA-seq datasets generated for this study have been deposited in the Sequence Read Archive (www.ncbi.nlm.nih.gov/sra) under the accession number PRJNA656429.

## Author Contributions

XZ, WX, and ZS designed this project. XZ and HY generated the transcriptome data and annotations. MS calculated the co-expression network. MS and JS completed the database construction and draft. All authors read and approved the final manuscript.

## Conflict of Interest

The authors declare that the research was conducted in the absence of any commercial or financial relationships that could be construed as a potential conflict of interest.

## Footnotes

1 http://bioinformatics.cau.edu.cn/HpeNet/

2 https://ftp.ncbi.nlm.nih.gov/blast/db/FASTA/

3 http://pfam.xfam.org/

4 http://www.ncbi.nlm.nih.gov/COG/

5 https://www.ebi.ac.uk/uniprot/

6 https://www.genome.jp/kegg/

7 http://geneontology.org/

8 http://pfam.xfam.org/search#tabview=tab3

9 https://www.arabidopsis.org/

10 https://www.apache.org/

11 https://www.mysql.com/

12 https://php.net/

13 http://js.cytoscape.org/
