# Supplementary material for: HpeNet: Co-expression Network Database for de novo Transcriptome Assembly of Paeonia lactiflora Pall
Source: Front Genet. 2020 Oct 21;11:570138. doi: 10.3389/fgene.2020.570138 (PMC7641121; doi:10.3389/fgene.2020.570138)
Supplement: Supplementary file 1 [file Data_Sheet_1.pdf]

frontiers

HpeNet: Co-expression Network Database for *De Novo* Transcriptome Assembly of *Paeonia lactiora* Pall

Minghao Sheng[1,2†], Jiajie She[2†], Wenying Xu[2], Yan Hong[1], Zhen Su[2*] and Xiaodong Zhang[1*]

[1] Beijing Agricultural Biotechnology Research Center, Beijing Engineering Research Center of Functional Floriculture, Beijing Academy of Agriculture and Forestry Science, Beijing, China, [2] State Key Laboratory of Plant Physiology and Biochemistry, College of Biological Sciences, China Agricultural University, Beijing, China

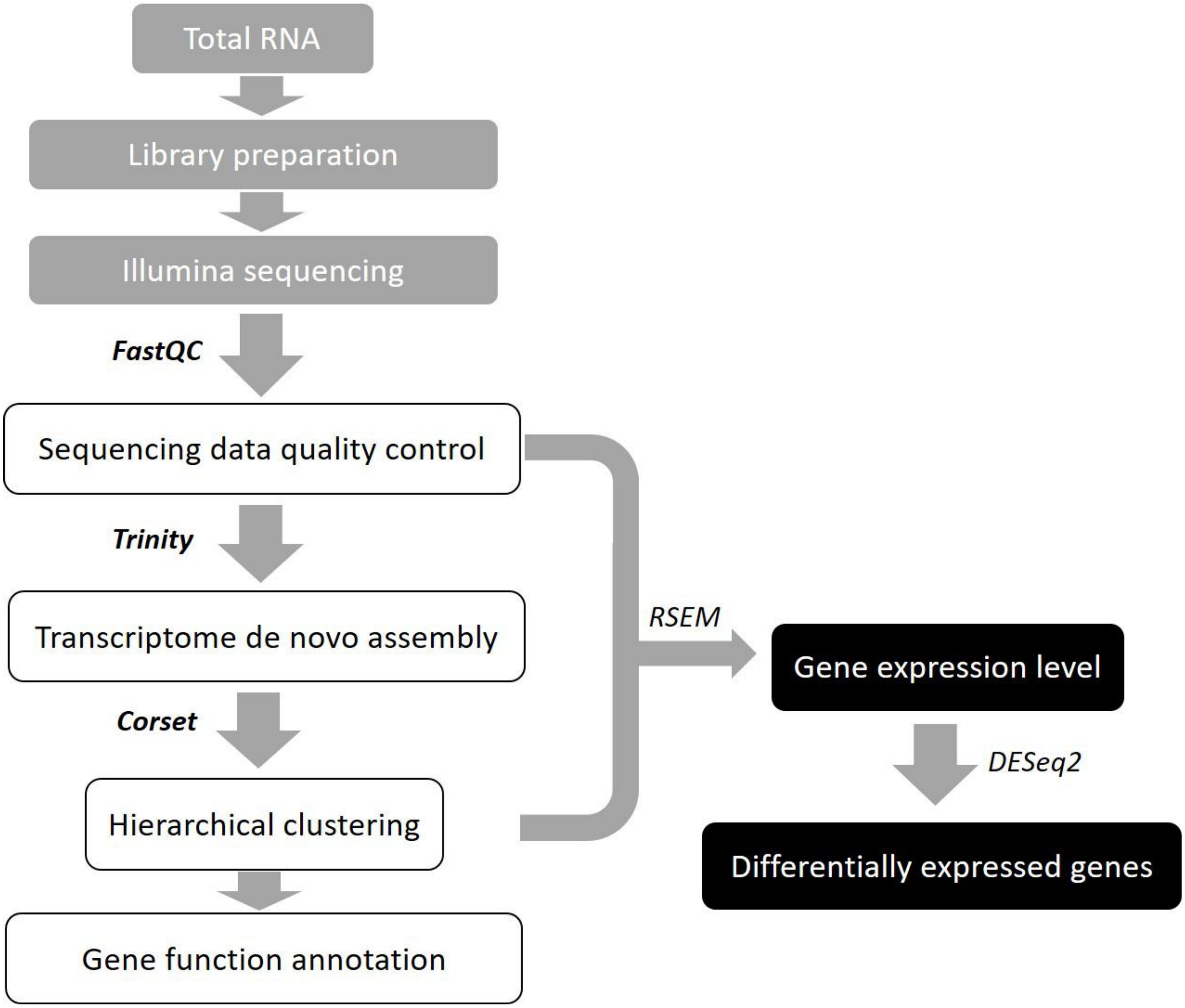

Figure S1. Flowchart showing steps in *Paeonia lactiflora* transcriptome assembly and annotation, downstream transcript clustering, and differential expression analyses.

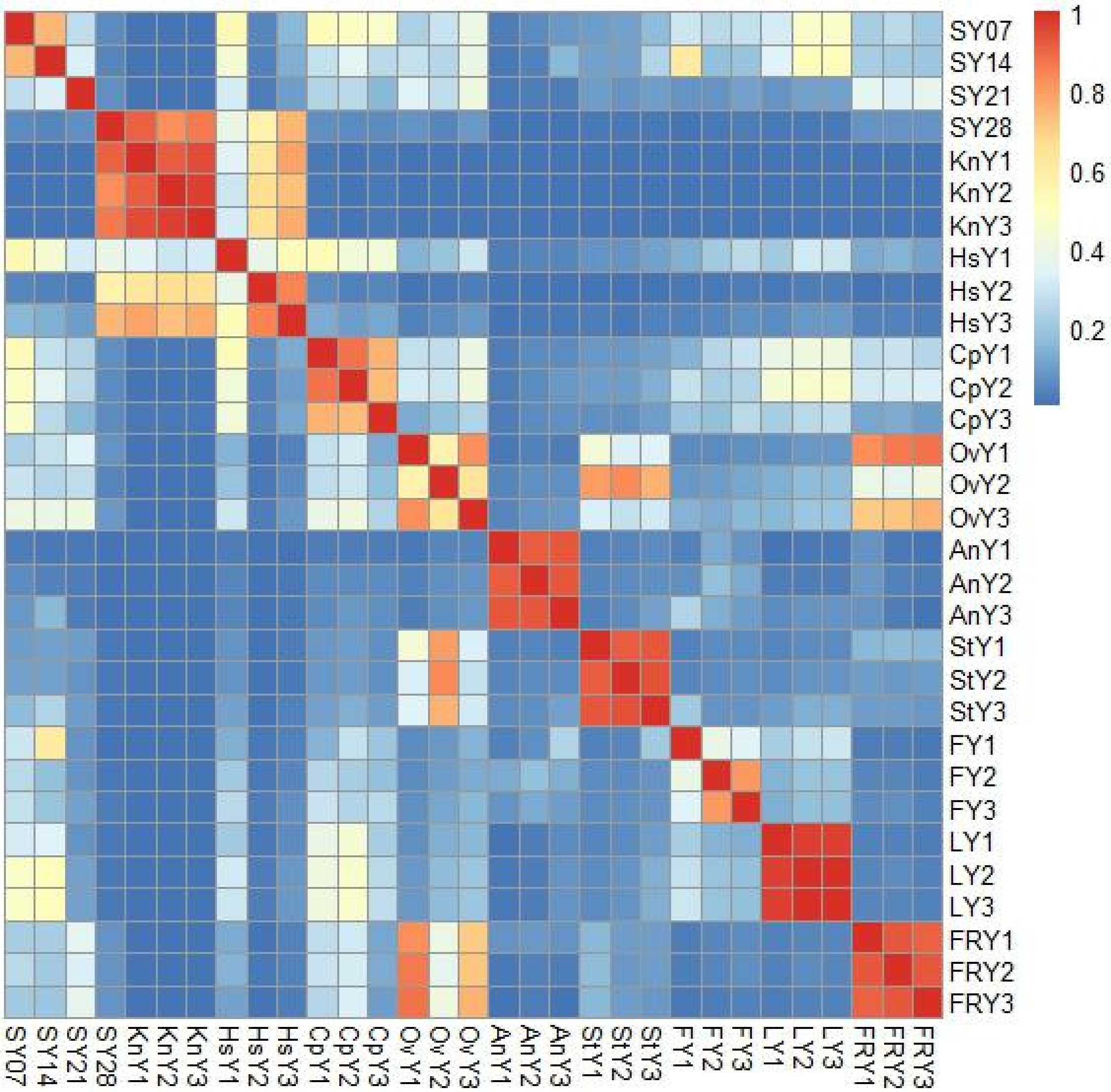

Figure S2. Correlation of expression patterns among the RNA-seq libraries for *Paeonia lactiflora* Pall. cv Shaoyou17C.

The red (high) and blue (low) colors represent the correlation of the samples. The analyses were performed using comparing the values of the entire transcriptome. Correlation analyses were performed using R software.

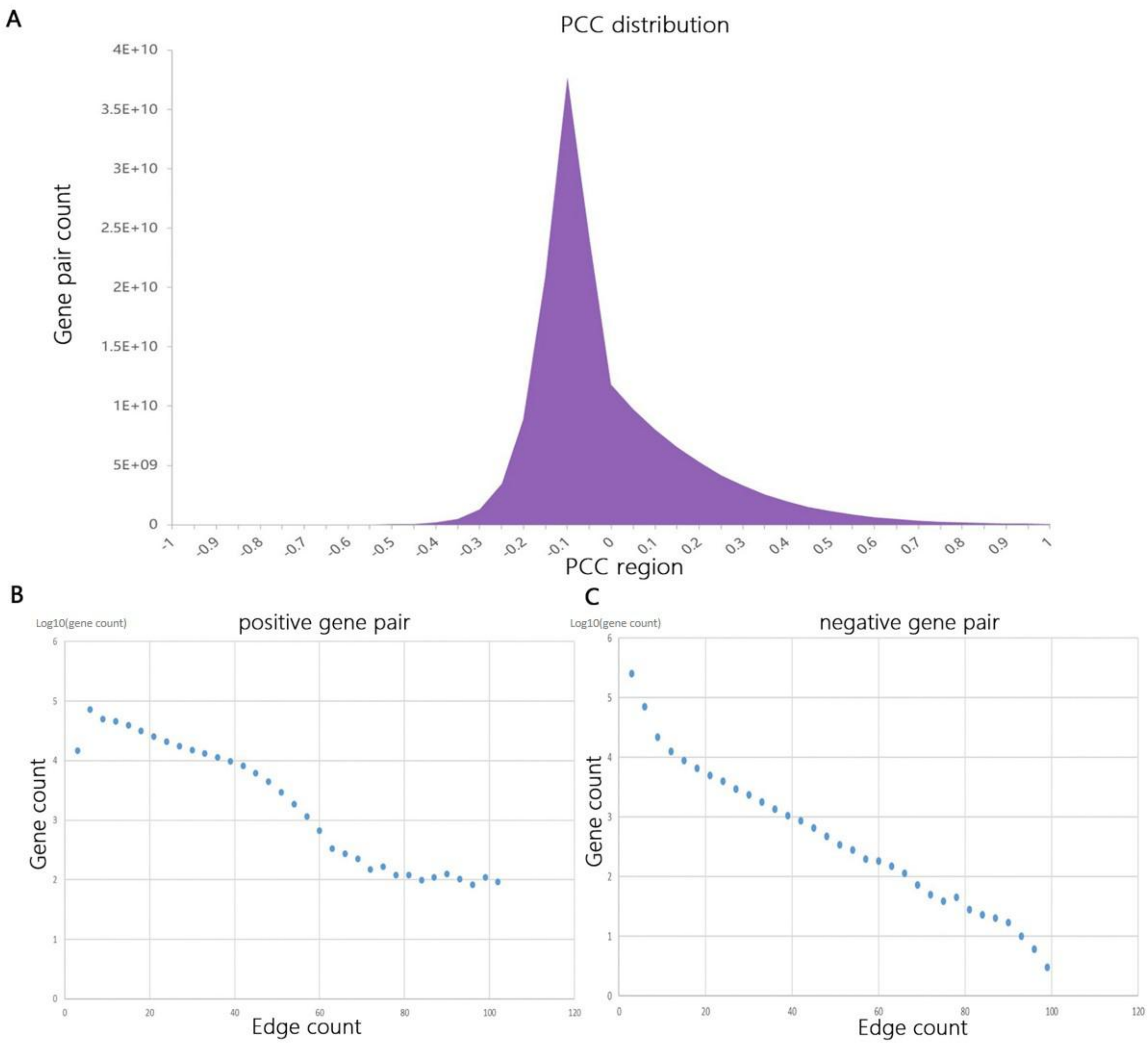

Figure S3. Results of co-expression network construction.

(A) Distribution of Pearson's correlation coefficient. Statistics for unigenes and gene pairs for the positive co-expression network **(B)** and the negative co-expression network **(C)**, respectively. These conform to the characteristics of biological network.

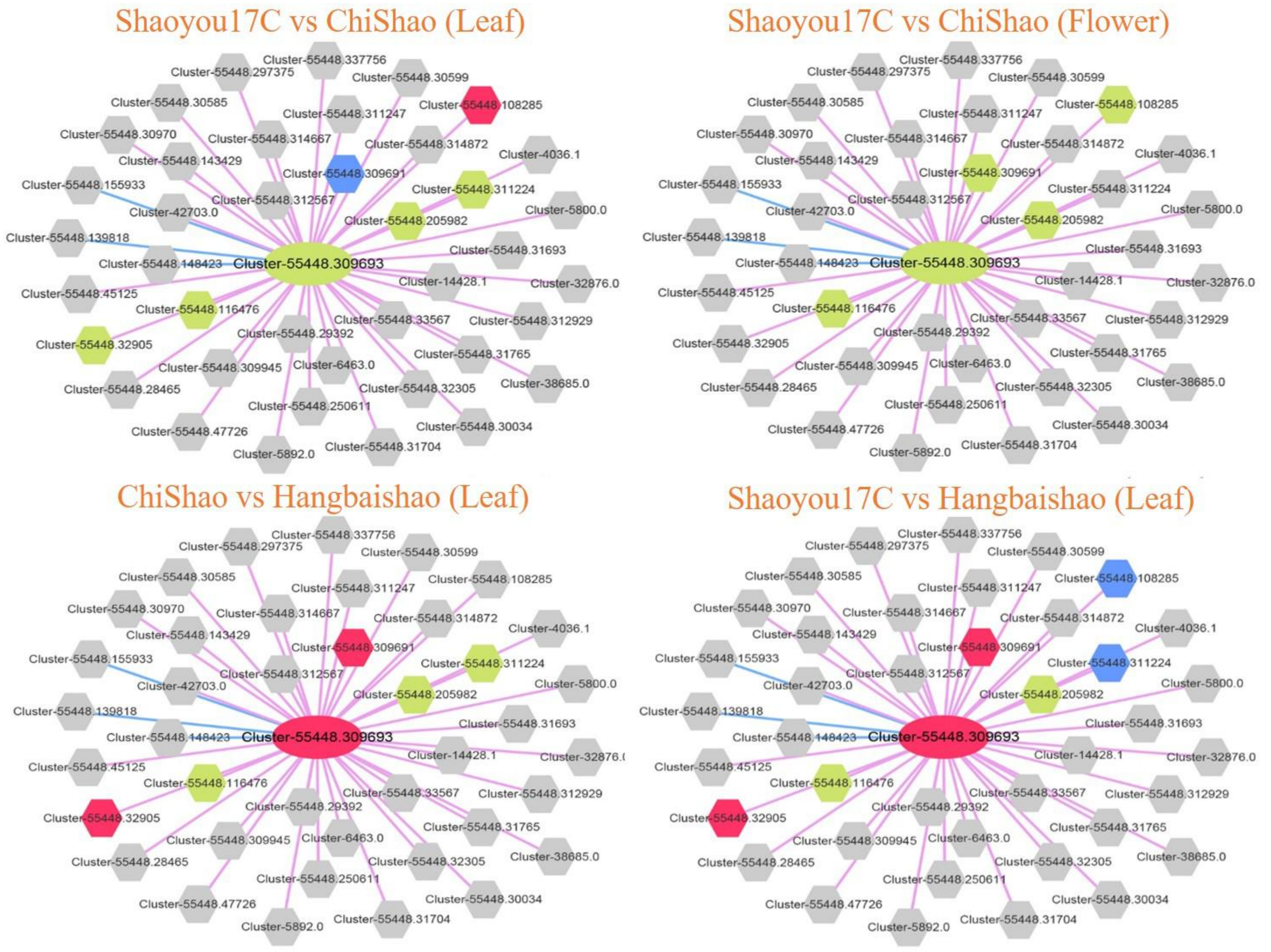

Figure S4. Expression view for differential expression of Cluster-55448.309693 (*FAD2*) co-expressed genes in different cultivars.

Genes expressed differentially between different cultivars that appeared in the same co-expression network as the query gene. The up-regulated genes in the front tissue are highlighted with red circles. The down-regulated genes in the front tissue are highlighted with blue circles. Co-expressed genes are highlighted with green circles. Gray circles represent genes without an expression profile in the given tissue.

Table S1. Sample information and data quality

Sample	Cultivar	Tissue	Clean Bases	Error(%)	Q20(%)	Q30(%)	GC Content(%)
**LY_1**	Shaoyou17C	Leaf	9.05G	0.02	96.89	92.07	44.71
**LY_2**	Shaoyou17C	Leaf	9.54G	0.02	96.82	91.90	45.09
**LY_3**	Shaoyou17C	Leaf	8.91G	0.02	96.13	90.50	44.96
**FY_1**	Shaoyou17C	Flower	8.54G	0.01	97.80	94.28	44.69
**FY_2**	Shaoyou17C	Flower	12.84G	0.02	96.56	91.40	44.10
**FY_3**	Shaoyou17C	Flower	9.05G	0.01	97.96	94.58	44.02
**SY_07**	Shaoyou17C	Seed(DAF7)	9.22G	0.02	96.28	90.76	44.52
**SY_14**	Shaoyou17C	Seed(DAF14)	9.83G	0.02	96.83	91.85	44.96
**SY_21**	Shaoyou17C	Seed(DAF21)	9.39G	0.02	96.18	90.90	44.75
**SY_28**	Shaoyou17C	Seed(DAF28)	9.86G	0.02	96.55	91.32	46.33
**OvY1**	Shaoyou17C	Ovary	8.43G	0.03	96.51	90.57	44.62
**OvY2**	Shaoyou17C	Ovary	8.61G	0.03	96.71	90.96	44.79
**OvY3**	Shaoyou17C	Ovary	7.74G	0.03	96.55	90.63	45.10
**StY1**	Shaoyou17C	Stigma	9.17G	0.03	96.77	91.07	44.87
**StY2**	Shaoyou17C	Stigma	8.54G	0.03	96.58	90.69	44.82
**StY3**	Shaoyou17C	Stigma	7.79G	0.03	96.73	90.96	44.64
**AnY1**	Shaoyou17C	Androecium	7.71G	0.03	96.58	90.73	45.31
**AnY2**	Shaoyou17C	Androecium	8.15G	0.03	96.72	91.02	45.35
**AnY3**	Shaoyou17C	Androecium	8.42G	0.03	96.68	90.91	45.22
**FRY1**	Shaoyou17C	Fibrous Root	7.29G	0.03	96.49	90.63	44.70
**FRY2**	Shaoyou17C	Fibrous Root	9.36G	0.03	96.60	90.80	44.57
**FRY3**	Shaoyou17C	Fibrous Root	7.58G	0.03	96.67	90.95	44.81
**CpY1**	Shaoyou17C	Pod	8.44G	0.02	98.76	95.90	45.14
**CpY2**	Shaoyou17C	Pod	9.63G	0.02	98.73	95.87	44.95
**CpY3**	Shaoyou17C	Pod	6.85G	0.02	98.69	95.85	44.96
**HsY1**	Shaoyou17C	Seed Coat	8.34G	0.02	98.67	95.69	44.89
**HsY2**	Shaoyou17C	Seed Coat	8.15G	0.02	98.69	95.84	45.78
**HsY3**	Shaoyou17C	Seed Coat	8.63G	0.02	98.79	95.98	45.42
**KnY1**	Shaoyou17C	kernel	6.81G	0.02	98.89	96.34	47.56
**KnY2**	Shaoyou17C	kernel	10.52G	0.03	97.29	92.70	47.74
**KnY3**	Shaoyou17C	kernel	7.17G	0.03	97.00	92.09	47.41
**LW_1**	Chishao	Leaf	8.40G	0.01	98.05	94.75	44.97
**LW_2**	Chishao	Leaf	10.01G	0.01	97.94	94.49	44.97
**LW_3**	Chishao	Leaf	8.87G	0.01	98.02	94.63	44.95
**FW_1**	Chishao	Flower	10.15G	0.01	97.89	94.45	44.03
**FW_2**	Chishao	Flower	9.17G	0.01	97.79	94.15	44.21
**FW_3**	Chishao	Flower	8.97G	0.01	97.66	93.86	43.91
**LMS_1**	Hangbaishao	Leaf	7.90G	0.01	98.00	94.61	44.97
**LMS_2**	Hangbaishao	Leaf	7.81G	0.01	98.05	94.72	44.94
**LMS_3**	Hangbaishao	Leaf	8.21G	0.01	98.03	94.70	44.69

Table S2. Assembly statistics for *Paeonia lactiflora* transcriptomes

Min Length	MeanLength	Median Length	Max Length	N50	N90	Total Nucleotides
201	965	624	13,684	1,462	418	401,336,869

Table S3. Number of unigenes annotated with KEGG

Pathway Hierarchy 1	Pathway Hierarchy 2	Gene Number
Organismal Systems	Environmental adaptation	2675
Cellular Processes	Transport and catabolism	3910
Environmental Information Processing	Membrane transport	589
Environmental Information Processing	Signal transduction	1908
Genetic Information Processing	Replication and repair	1966
Genetic Information Processing	Transcription	2727
Genetic Information Processing	Folding, sorting and degradation	5863
Genetic Information Processing	Translation	8186
Metabolism	Glycan biosynthesis and metabolism	1182
Metabolism	Metabolism of terpenoids and polyketides	1725
Metabolism	Metabolism of cofactors and vitamins	1787
Metabolism	Biosynthesis of other secondary metabolites	2282
Metabolism	Metabolism of other amino acids	2300
Metabolism	Nucleotide metabolism	2930
Metabolism	Energy metabolism	3576
**Metabolism**	**Lipid metabolism**	**4880**
Metabolism	Amino acid metabolism	5215
Metabolism	Overview	6660
Metabolism	Carbohydrate metabolism	10494

Table S4. Number of unigenes annotated with GO

GO term	Description	Category	Unigene Number
GO:0009987	cellular process	Biological Process	77134
GO:0008152	metabolic process	Biological Process	72426
GO:0044699	single-organism process	Biological Process	56888
GO:0065007	biological regulation	Biological Process	25177
GO:0050789	regulation of biological process	Biological Process	23229
GO:0051179	localization	Biological Process	21919
GO:0050896	response to stimulus	Biological Process	17932
GO:0071840	cellular component organization or biogenesis	Biological Process	14589
GO:0023052	signaling	Biological Process	8912
GO:0051704	multi-organism process	Biological Process	7469
GO:0032501	multicellular organismal process	Biological Process	3556
GO:0032502	developmental process	Biological Process	2930
GO:0000003	reproduction	Biological Process	1810
GO:0022414	reproductive process	Biological Process	1681
GO:0048518	positive regulation of biological process	Biological Process	1543
GO:0048519	negative regulation of biological process	Biological Process	1343
GO:0040011	locomotion	Biological Process	1252
GO:0002376	immune system process	Biological Process	1241
GO:0022610	biological adhesion	Biological Process	993
GO:0040007	growth	Biological Process	292
GO:0007610	behavior	Biological Process	175
GO:0001906	cell killing	Biological Process	153
GO:0098754	detoxification	Biological Process	106
GO:0044848	biological phase	Biological Process	105
GO:0048511	rhythmic process	Biological Process	63
GO:0098743	cell aggregation	Biological Process	8
GO:0005623	cell	Cellular Component	43915
GO:0044464	cell part	Cellular Component	43890
GO:0043226	organelle	Cellular Component	30149
GO:0032991	macromolecular complex	Cellular Component	26460
GO:0016020	membrane	Cellular Component	23101
GO:0044425	membrane part	Cellular Component	20741
GO:0044422	organelle part	Cellular Component	14518
GO:0031974	membrane-enclosed lumen	Cellular Component	4072
GO:0044423	virion part	Cellular Component	2985
GO:0019012	virion	Cellular Component	2985
GO:0005576	extracellular region	Cellular Component	1498
GO:0044421	extracellular region part	Cellular Component	1218
GO:0044217	other organism part	Cellular Component	856
GO:0044215	other organism	Cellular Component	856

GO:0031012	extracellular matrix	Cellular Component	413
GO:0030054	cell junction	Cellular Component	145
GO:0055044	symplast	Cellular Component	50
GO:0044456	synapse part	Cellular Component	22
GO:0045202	synapse	Cellular Component	22
GO:0044420	extracellular matrix component	Cellular Component	9
GO:0009295	nucleoid	Cellular Component	5
GO:0005488	binding	Molecular Function	75385
GO:0003824	catalytic activity	Molecular Function	60667
GO:0005215	transporter activity	Molecular Function	9394
GO:0005198	structural molecule activity	Molecular Function	4950
GO:0001071	nucleic acid binding transcription factor activity	Molecular Function	3716
GO:0098772	molecular function regulator	Molecular Function	3034
GO:0060089	molecular transducer activity	Molecular Function	2476
GO:0000988	transcription factor activity, protein binding	Molecular Function	1263
GO:0016209	antioxidant activity	Molecular Function	641
GO:0016530	metallochaperone activity	Molecular Function	40

Table S5. Mapping results for mRNA-seq

Sample	Raw Reads	Clean Reads	Total Mapped Reads	Mapping Rate(%)
**LY_1**	60340464	60340464	50784418	84.16
**LY_2**	63589422	63589422	53671182	84.40
**LY_3**	59414578	59414578	50308530	84.67
**FY_1**	56936480	56936480	48125926	84.53
**FY_2**	85572774	85572774	71388532	83.15
**FY_3**	60348118	60348118	50812032	84.20
**SY_07**	61460356	61460356	48229978	78.47
**SY_14**	65565528	65565528	51462812	78.49
**SY_21**	62618112	62618112	49903822	79.70
**SY_28**	65743100	65743100	49128326	74.73
**OvY1**	57286942	56209582	46346810	82.45
**OvY2**	58523018	57422184	47022886	81.89
**OvY3**	52423944	51588788	43285184	83.90
**StY1**	62279826	61110896	47552104	77.81
**StY2**	58075044	56912620	44335016	77.90
**StY3**	53561382	51958968	41492084	79.67
**AnY1**	52670966	51390036	40103530	78.04
**AnY2**	55957872	54339336	43584694	80.21
**AnY3**	57723216	56150288	43984372	78.33
**FRY1**	50478086	48594730	38815500	79.88
**FRY2**	64634766	62432484	50429244	80.77
**FRY3**	52106454	50550620	41082228	81.27
**CpY1**	57539586	56293282	47382752	84.17
**CpY2**	65795476	64191554	53526232	83.39
**CpY3**	47841496	45661590	37559010	82.26
**HsY1**	56579918	55570140	46678612	84.00
**HsY2**	55643630	54355424	42485704	78.16
**HsY3**	59017892	57560356	46832526	81.36
**KnY1**	46445836	45380580	32729388	72.12
**KnY2**	71318114	70151592	48310098	68.87
**KnY3**	48766252	47824456	33981196	70.85
**LW_1**	56007438	56007438	46199014	82.49
**LW_2**	66734360	66734360	55115146	82.59
**LW_3**	59162746	59162746	49095766	82.98
**FW_1**	67689686	67689686	54986528	81.23
**FW_2**	61165712	61165712	48601764	79.46
**FW_3**	59796530	59796530	49096166	82.11
**LMS_1**	52670898	52670898	43434370	82.46
**LMS_2**	52059704	52059704	43099768	82.79
**LMS_3**	54752454	54752454	44749836	81.73

Table S6. Number of DEGs between different cultivars

Samples	Tissue	DEGs		up-regulated		down-regulated	
		|log2FoldChange| > 1	|log2FoldChange| > 2	FoldChange > 2	FoldChange > 4	FoldChange < 0.5	FoldChange < 0.25
**LY VS LW**	Leaf	46,117	33,910	25,404	20,104	20,713	13,806
**LY VS LMS**	Leaf	42,873	30,164	28,299	20,606	14,574	9,558
**LW VS LMS**	Leaf	55,685	43,517	37,043	29,865	18,642	13,652
**FY VS FW**	Flower	25,179	17,734	8,266	5,791	16,913	11,943

Table S7. Unigenes for light-harvesting complex photosystems I and II in *Paeonia lactiflora*

Genes	AGI	E-value	Ara_symbol	Annotation
Cluster-55448.185067	AT3G54890	4.8E-17	LHCA1	photosystem I light harvesting complex gene 1
Cluster-55448.254675	AT3G54890	9.7E-18	LHCA1	photosystem I light harvesting complex gene 1
Cluster-8750.0	AT3G54890	1.3E-14	LHCA1	photosystem I light harvesting complex gene 1
Cluster-10922.0	AT3G54890	2.1E-18	LHCA1	photosystem I light harvesting complex gene 1
Cluster-50825.0	AT3G54890	9.9E-07	LHCA1	photosystem I light harvesting complex gene 1
Cluster-55448.299356	AT3G54890	8.1E-17	LHCA1	photosystem I light harvesting complex gene 1
Cluster-10523.0	AT3G54890	1.6E-15	LHCA1	photosystem I light harvesting complex gene 1
Cluster-43507.0	AT3G54890	4.9E-16	LHCA1	photosystem I light harvesting complex gene 1
Cluster-55448.255790	AT3G54890	5.7E-18	LHCA1	photosystem I light harvesting complex gene 1
Cluster-55448.169583	AT3G54890	7.5E-15	LHCA1	photosystem I light harvesting complex gene 1
Cluster-49302.4	AT3G54890	4.8E-13	LHCA1	photosystem I light harvesting complex gene 1
Cluster-49302.3	AT3G54890	7.3E-10	LHCA1	photosystem I light harvesting complex gene 1
Cluster-49302.1	AT3G54890	2E-19	LHCA1	photosystem I light harvesting complex gene 1
Cluster-55448.254668	AT3G54890	7.7E-18	LHCA1	photosystem I light harvesting complex gene 1
Cluster-54169.0	AT3G54890	2E-18	LHCA1	photosystem I light harvesting complex gene 1
Cluster-55448.43366	AT3G54890	1.9E-12	LHCA1	photosystem I light harvesting complex gene 1
Cluster-55448.43365	AT3G54890	2.8E-14	LHCA1	photosystem I light harvesting complex gene 1
Cluster-55448.233148	AT3G54890	1.8E-32	LHCA1	photosystem I light harvesting complex gene 1
Cluster-55448.207014	AT3G54890	1E-17	LHCA1	photosystem I light harvesting complex gene 1
Cluster-55448.124893	AT3G54890	2.2E-14	LHCA1	photosystem I light harvesting complex gene 1
Cluster-55448.165048	AT3G54890	4E-17	LHCA1	photosystem I light harvesting complex gene 1
Cluster-54037.0	AT3G54890	3.5E-18	LHCA1	photosystem I light harvesting complex gene 1
Cluster-55448.116224	AT3G54890	3.5E-07	LHCA1	photosystem I light harvesting complex gene 1
Cluster-55448.162486	AT3G54890	4.1E-17	LHCA1	photosystem I light harvesting complex gene 1
Cluster-55448.238335	AT3G54890	3.1E-17	LHCA1	photosystem I light harvesting complex gene 1
Cluster-55448.254672	AT3G61470	3.7E-32	LHCA2	photosystem I light harvesting complex gene 2
Cluster-55448.254674	AT3G61470	5.5E-29	LHCA2	photosystem I light harvesting complex gene 2
Cluster-55448.194212	AT3G61470	3.8E-24	LHCA2	photosystem I light harvesting complex gene 2
Cluster-55448.254667	AT3G61470	0.0000065	LHCA2	photosystem I light harvesting complex gene 2
Cluster-46216.0	AT3G61470	0.0000022	LHCA2	photosystem I light harvesting complex gene 2
Cluster-55448.90391	AT3G61470	1.4E-29	LHCA2	photosystem I light harvesting complex gene 2
Cluster-55448.148699	AT3G61470	2.9E-24	LHCA2	photosystem I light harvesting complex gene 2
Cluster-55448.208985	AT3G61470	1.8E-29	LHCA2	photosystem I light harvesting complex gene 2
Cluster-55448.208986	AT3G61470	2E-29	LHCA2	photosystem I light harvesting complex gene 2
Cluster-55448.208983	AT3G61470	2E-29	LHCA2	photosystem I light harvesting complex gene 2
Cluster-42805.0	AT3G61470	4.6E-32	LHCA2	photosystem I light harvesting complex gene 2
Cluster-55448.174063	AT3G61470	3.6E-33	LHCA2	photosystem I light harvesting complex gene 2
Cluster-10591.0	AT3G61470	3.1E-31	LHCA2	photosystem I light harvesting complex gene 2
Cluster-48665.0	AT3G61470	1.7E-30	LHCA2	photosystem I light harvesting complex gene 2
Cluster-52182.0	AT3G61470	1.1E-24	LHCA2	photosystem I light harvesting complex gene 2
Cluster-55448.165047	AT3G61470	3.7E-17	LHCA2	photosystem I light harvesting complex gene 2

Cluster-54169.2	AT3G61470	1.8E-31	LHCA2	photosystem I light harvesting complex gene 2
Cluster-55448.208984	AT3G61470	2E-29	LHCA2	photosystem I light harvesting complex gene 2
Cluster-55448.180252	AT3G61470	3.7E-33	LHCA2	photosystem I light harvesting complex gene 2
Cluster-55448.254669	AT3G61470	6.1E-18	LHCA2	photosystem I light harvesting complex gene 2
Cluster-55448.180251	AT3G61470	3.8E-33	LHCA2	photosystem I light harvesting complex gene 2
Cluster-55448.180250	AT3G61470	3.2E-33	LHCA2	photosystem I light harvesting complex gene 2
Cluster-55448.41270	AT1G61520	1.1E-31	LHCA3	photosystem I light harvesting complex gene 3
Cluster-53735.0	AT1G61520	9.8E-15	LHCA3	photosystem I light harvesting complex gene 3
Cluster-55448.290535	AT1G61520	2.9E-10	LHCA3	photosystem I light harvesting complex gene 3
Cluster-43042.0	AT1G61520	2.7E-33	LHCA3	photosystem I light harvesting complex gene 3
Cluster-55448.154768	AT1G61520	1.2E-24	LHCA3	photosystem I light harvesting complex gene 3
Cluster-55448.12570	AT1G61520	4.9E-30	LHCA3	photosystem I light harvesting complex gene 3
Cluster-55448.329554	AT1G61520	2E-31	LHCA3	photosystem I light harvesting complex gene 3
Cluster-55448.299334	AT1G61520	1.9E-31	LHCA3	photosystem I light harvesting complex gene 3
Cluster-55448.299274	AT1G61520	1.9E-31	LHCA3	photosystem I light harvesting complex gene 3
Cluster-55448.291948	AT1G61520	3.1E-10	LHCA3	photosystem I light harvesting complex gene 3
Cluster-54169.1	AT1G61520	5.5E-16	LHCA3	photosystem I light harvesting complex gene 3
Cluster-48200.0	AT1G61520	5E-26	LHCA3	photosystem I light harvesting complex gene 3
Cluster-63658.0	AT1G61520	1.8E-28	LHCA3	photosystem I light harvesting complex gene 3
Cluster-53552.0	AT1G61520	7.8E-30	LHCA3	photosystem I light harvesting complex gene 3
Cluster-63878.0	AT1G61520	8.5E-29	LHCA3	photosystem I light harvesting complex gene 3
Cluster-55448.322094	AT1G61520	8E-32	LHCA3	photosystem I light harvesting complex gene 3
Cluster-55448.221545	AT1G61520	1.1E-13	LHCA3	photosystem I light harvesting complex gene 3
Cluster-55448.190217	AT1G61520	3.3E-29	LHCA3	photosystem I light harvesting complex gene 3
Cluster-64199.1	AT1G61520	3.1E-24	LHCA3	photosystem I light harvesting complex gene 3
Cluster-64199.0	AT1G61520	3E-24	LHCA3	photosystem I light harvesting complex gene 3
Cluster-55448.124892	AT1G61520	6E-31	LHCA3	photosystem I light harvesting complex gene 3
Cluster-62353.0	AT1G61520	2.6E-24	LHCA3	photosystem I light harvesting complex gene 3
Cluster-56178.0	AT1G61520	1.3E-31	LHCA3	photosystem I light harvesting complex gene 3
Cluster-55448.254673	AT1G45474	5.1E-32	Lhca5	photosystem I light harvesting complex gene 5
Cluster-55448.254677	AT1G45474	4.7E-32	Lhca5	photosystem I light harvesting complex gene 5
Cluster-55448.204548	AT1G45474	3.4E-11	Lhca5	photosystem I light harvesting complex gene 5
Cluster-55448.30131	AT1G45474	4.2E-11	Lhca5	photosystem I light harvesting complex gene 5
Cluster-55448.90389	AT1G45474	2.9E-27	Lhca5	photosystem I light harvesting complex gene 5
Cluster-55448.164933	AT1G45474	2.2E-31	Lhca5	photosystem I light harvesting complex gene 5
Cluster-43157.0	AT1G45474	2.1E-21	Lhca5	photosystem I light harvesting complex gene 5
Cluster-55448.90390	AT1G45474	4.6E-29	Lhca5	photosystem I light harvesting complex gene 5
Cluster-55448.64351	AT1G45474	6E-09	Lhca5	photosystem I light harvesting complex gene 5
Cluster-47940.0	AT1G45474	4.7E-09	Lhca5	photosystem I light harvesting complex gene 5
Cluster-55448.318	AT1G45474	8.1E-32	Lhca5	photosystem I light harvesting complex gene 5
Cluster-55448.319	AT1G45474	1.5E-31	Lhca5	photosystem I light harvesting complex gene 5
Cluster-55448.239466	AT1G45474	3.4E-07	Lhca5	photosystem I light harvesting complex gene 5
Cluster-55448.239465	AT1G45474	8.1E-08	Lhca5	photosystem I light harvesting complex gene 5

Cluster-55448.239464	AT1G45474	3.3E-07	Lhca5	photosystem I light harvesting complex gene 5
Cluster-55448.154574	AT1G45474	3.9E-11	Lhca5	photosystem I light harvesting complex gene 5
Cluster-61642.0	AT1G45474	3E-32	Lhca5	photosystem I light harvesting complex gene 5
Cluster-55448.312809	AT1G45474	1.2E-09	Lhca5	photosystem I light harvesting complex gene 5
Cluster-55448.312803	AT1G45474	1.1E-09	Lhca5	photosystem I light harvesting complex gene 5
Cluster-55448.312805	AT1G45474	3.4E-13	Lhca5	photosystem I light harvesting complex gene 5
Cluster-55448.220405	AT1G45474	1.3E-09	Lhca5	photosystem I light harvesting complex gene 5
Cluster-55448.182455	AT1G45474	2E-12	Lhca5	photosystem I light harvesting complex gene 5
Cluster-55448.277968	AT1G45474	0.0000071	Lhca5	photosystem I light harvesting complex gene 5
Cluster-52799.0	AT1G45474	8.6E-09	Lhca5	photosystem I light harvesting complex gene 5
Cluster-55448.301166	AT1G45474	9.1E-09	Lhca5	photosystem I light harvesting complex gene 5
Cluster-55448.165049	AT1G45474	4.3E-11	Lhca5	photosystem I light harvesting complex gene 5
Cluster-55448.52830	AT1G45474	2.9E-07	Lhca5	photosystem I light harvesting complex gene 5
Cluster-55448.33151	AT1G45474	4.3E-10	Lhca5	photosystem I light harvesting complex gene 5
Cluster-55448.165050	AT1G45474	3.5E-11	Lhca5	photosystem I light harvesting complex gene 5
Cluster-55448.67687	AT1G45474	7.2E-09	Lhca5	photosystem I light harvesting complex gene 5
Cluster-55448.67686	AT1G45474	1.4E-11	Lhca5	photosystem I light harvesting complex gene 5
Cluster-59974.0	AT1G45474	1.5E-31	Lhca5	photosystem I light harvesting complex gene 5
Cluster-55448.145353	AT1G45474	2.3E-12	Lhca5	photosystem I light harvesting complex gene 5
Cluster-42160.0	AT1G45474	8E-29	Lhca5	photosystem I light harvesting complex gene 5
Cluster-54169.3	AT1G45474	6.7E-26	Lhca5	photosystem I light harvesting complex gene 5
Cluster-44575.0	AT1G45474	1.9E-29	Lhca5	photosystem I light harvesting complex gene 5
Cluster-42553.0	AT1G45474	6.6E-31	Lhca5	photosystem I light harvesting complex gene 5
Cluster-55448.125390	AT1G45474	3.9E-07	Lhca5	photosystem I light harvesting complex gene 5
Cluster-55448.239481	AT1G45474	0.0000001	Lhca5	photosystem I light harvesting complex gene 5
Cluster-55448.207446	AT1G45474	8.7E-24	Lhca5	photosystem I light harvesting complex gene 5
Cluster-15173.0	AT1G45474	7E-13	Lhca5	photosystem I light harvesting complex gene 5
Cluster-55448.256570	AT1G45474	7.6E-09	Lhca5	photosystem I light harvesting complex gene 5
Cluster-55448.315057	AT1G45474	4E-11	Lhca5	photosystem I light harvesting complex gene 5
Cluster-55448.315059	AT1G45474	1.1E-09	Lhca5	photosystem I light harvesting complex gene 5
Cluster-55448.136777	AT1G45474	3.9E-07	Lhca5	photosystem I light harvesting complex gene 5
Cluster-55448.300294	AT1G45474	1.1E-31	Lhca5	photosystem I light harvesting complex gene 5
Cluster-55448.300144	AT1G45474	2.9E-12	Lhca5	photosystem I light harvesting complex gene 5
Cluster-55316.0	AT1G45474	1.6E-31	Lhca5	photosystem I light harvesting complex gene 5
Cluster-55448.66257	AT1G45474	5.9E-12	Lhca5	photosystem I light harvesting complex gene 5
Cluster-63847.0	AT1G45474	0.0000064	Lhca5	photosystem I light harvesting complex gene 5
Cluster-55448.123573	AT1G29930	1.4E-25	AB140	chlorophyll A/B binding protein 1
Cluster-55448.252165	AT1G29920	6E-08	AB165	chlorophyll A/B-binding protein 2
Cluster-10274.0	AT1G29920	4.4E-27	AB165	chlorophyll A/B-binding protein 2
Cluster-53555.0	AT1G29910	8.7E-23	AB180	chlorophyll A/B binding protein 3
Cluster-10556.0	AT1G29910	7.6E-20	AB180	chlorophyll A/B binding protein 3
Cluster-58517.0	AT1G29910	3.3E-26	AB180	chlorophyll A/B binding protein 3
Cluster-42989.0	AT1G29910	1.4E-26	AB180	chlorophyll A/B binding protein 3
Cluster-55448.205415	AT1G29910	6.5E-33	AB180	chlorophyll A/B binding protein 3

Cluster-55448.300813	AT1G29910	3.4E-21	AB180	chlorophyll A/B binding protein 3
Cluster-55448.196065	AT1G29910	5.7E-23	AB180	chlorophyll A/B binding protein 3
Cluster-55448.300800	AT1G29910	1.8E-33	AB180	chlorophyll A/B binding protein 3
Cluster-55448.82948	AT1G15820	0.0000037	CP24	light harvesting complex photosystem II subunit 6
Cluster-55448.207195	AT1G15820	6.1E-32	CP24	light harvesting complex photosystem II subunit 6
Cluster-50118.0	AT1G15820	3.8E-43	CP24	light harvesting complex photosystem II subunit 6
Cluster-10595.0	AT1G15820	0.0000067	CP24	light harvesting complex photosystem II subunit 6
Cluster-55448.178608	AT2G34430	5.4E-07	LHB1B1	light-harvesting chlorophyll-protein complex II subunit B1
Cluster-55448.252164	AT2G34430	5.6E-14	LHB1B1	light-harvesting chlorophyll-protein complex II subunit B1
Cluster-55448.200801	AT2G34430	1.1E-27	LHB1B1	light-harvesting chlorophyll-protein complex II subunit B1
Cluster-55448.139993	AT2G34420	3.3E-23	LHB1B2	photosystem II light harvesting complex gene B1B2
Cluster-55448.90386	AT2G34420	4.5E-26	LHB1B2	photosystem II light harvesting complex gene B1B2
Cluster-10505.1	AT2G34420	9.7E-07	LHB1B2	photosystem II light harvesting complex gene B1B2
Cluster-30989.0	AT2G34420	4E-28	LHB1B2	photosystem II light harvesting complex gene B1B2
Cluster-55448.232765	AT2G34420	6.6E-30	LHB1B2	photosystem II light harvesting complex gene B1B2
Cluster-55448.253390	AT2G05070	2.5E-21	LHCB2	photosystem II light harvesting complex gene 2.2
Cluster-55448.254671	AT2G05070	9E-08	LHCB2	photosystem II light harvesting complex gene 2.2
Cluster-55448.177689	AT2G05070	3E-26	LHCB2	photosystem II light harvesting complex gene 2.2
Cluster-55448.180380	AT2G05070	3.1E-26	LHCB2	photosystem II light harvesting complex gene 2.2
Cluster-48948.1	AT2G05070	4E-10	LHCB2	photosystem II light harvesting complex gene 2.2
Cluster-64130.0	AT2G05070	3.4E-14	LHCB2	photosystem II light harvesting complex gene 2.2
Cluster-42856.0	AT2G05100	5.6E-09	LHCB2	photosystem II light harvesting complex gene 2.1
Cluster-41662.0	AT2G05100	2.3E-30	LHCB2	photosystem II light harvesting complex gene 2.1
Cluster-57027.0	AT2G05100	4.8E-10	LHCB2	photosystem II light harvesting complex gene 2.1
Cluster-10028.0	AT2G05100	6E-27	LHCB2	photosystem II light harvesting complex gene 2.1
Cluster-48948.0	AT2G05100	0.0000038	LHCB2	photosystem II light harvesting complex gene 2.1
Cluster-47318.0	AT2G05100	6.7E-31	LHCB2	photosystem II light harvesting complex gene 2.1
Cluster-55448.311650	AT3G27690	2.6E-26	LHCB2	photosystem II light harvesting complex gene 2.3
Cluster-42862.0	AT3G27690	1.9E-20	LHCB2	photosystem II light harvesting complex gene 2.3
Cluster-55448.149407	AT3G27690	4.3E-31	LHCB2	photosystem II light harvesting complex gene 2.3
Cluster-55448.12811	AT3G27690	4.1E-08	LHCB2	photosystem II light harvesting complex gene 2.3
Cluster-47940.1	AT3G27690	1.3E-22	LHCB2	photosystem II light harvesting complex gene 2.3
Cluster-36118.0	AT3G27690	7.1E-27	LHCB2	photosystem II light harvesting complex gene 2.3
Cluster-44237.0	AT5G54270	1.9E-18	LHCB3	light-harvesting chlorophyll B-binding protein 3
Cluster-55448.340052	AT5G54270	4.3E-19	LHCB3	light-harvesting chlorophyll B-binding protein 3
Cluster-55448.150632	AT5G54270	7.7E-18	LHCB3	light-harvesting chlorophyll B-binding protein 3
Cluster-49413.0	AT5G54270	2.8E-14	LHCB3	light-harvesting chlorophyll B-binding protein 3
Cluster-49302.2	AT5G54270	3.7E-09	LHCB3	light-harvesting chlorophyll B-binding protein 3
Cluster-55448.342166	AT5G54270	1.1E-08	LHCB3	light-harvesting chlorophyll B-binding protein 3
Cluster-50118.1	AT2G40100	2.3E-12	LHCB4.3	light harvesting complex photosystem II
Cluster-54070.0	AT2G40100	3E-11	LHCB4.3	light harvesting complex photosystem II
Cluster-10595.1	AT2G40100	0.0000049	LHCB4.3	light harvesting complex photosystem II

Cluster-48386.0	AT4G10340	5.3E-07	LHCB5	light harvesting complex of photosystem II 5
Cluster-55448.254676	AT4G10340	6.8E-08	LHCB5	light harvesting complex of photosystem II 5
Cluster-10441.0	AT4G10340	1.3E-12	LHCB5	light harvesting complex of photosystem II 5
Cluster-55448.198158	AT4G10340	3.3E-23	LHCB5	light harvesting complex of photosystem II 5
Cluster-52921.0	AT4G10340	0.0000085	LHCB5	light harvesting complex of photosystem II 5

Table S8. Expression profiles for unigenes in the FADs network

Gene_id	SY_07	SY_14	SY_21	SY_28	KnY-Median	HsY-Median	CpY-Median	LY-Median	FRY-Median
Cluster-55448.309693	0	0.61	3.45	363.12	166	1167.96	0.16	44.29	0
Cluster-55448.309691	0	0	0.1	0.89	0.91	3.58	0	0.75	0
Cluster-55448.32905	0.07	0.3	3.42	309.77	69.32	1017.03	0.32	34.97	0
Cluster-38685.0	0	0	0	0	0	1.53	0	0	0
Cluster-55448.29392	0	0.28	0	0.13	0	2.21	0	0	0
Cluster-42703.0	0	0	0	0	0	0.24	0	0	0
Cluster-55448.28465	0	0	0	0	0	0.04	0	0	0
Cluster-55448.250611	0.05	1.01	2.8	10.17	1.72	22.42	0	0	0
Cluster-32876.0	0	0	0	0	0	0.2	0	0	0
Cluster-55448.70214	0	0	0	0	0	0.08	0	0	0
Cluster-55448.143429	0	0	0	0	0	0.3	0	0	0
Cluster-55448.118765	0.2	0.38	0.36	0.11	0.32	1.59	0.1	0.59	0.67
Cluster-55448.312567	0	0	3.39	29.71	0	77.05	0	0	0
Cluster-55448.99355	0.29	0.05	0.06	0.17	0.06	0.59	0	0.05	0
Cluster-55448.30585	0	0	0	3.01	0.07	5.57	0	0	0
Cluster-55448.210681	0	0	0	0	0	0.07	0	0	0
Cluster-55448.31704	0	0	0	0.86	0	2.6	0	0	0.1
Cluster-54354.0	0	0	0	0.8	0.28	4.28	0	0	0
Cluster-55448.252811	0	0	0	0.06	0	0.31	0	0	0
Cluster-55448.51735	0	0	0	0	0	0.16	0	0	0
Cluster-55448.230922	0.15	0	0	0.22	0.22	0.26	0	0.2	0
Cluster-55448.311247	0	0	9.22	41.56	112.7	1221.6	0.23	0	0
Cluster-55448.76989	0	0.06	0	0.67	0.11	5.95	0	0	0
Cluster-55448.47726	0	0	0	0	0	1.99	0	0	0
Cluster-55448.314388	0	0	0	0.16	0	1.65	0	0	0
Cluster-55448.30970	0	0	0	1.26	0	5.96	0	0	0
Cluster-55448.232891	2.81	3.89	4.26	19.91	5.91	34.05	4.14	8.64	10.88
Cluster-55448.205982	0.02	1.17	3.19	14.33	1.77	13.24	0	0.04	0.02
Cluster-55448.89390	0.35	30.71	382.21	1652.63	32.82	2765.18	1.45	0	0
Cluster-55448.309421	0.35	31.75	316.82	654.78	18.4	1096	0.22	0	0
Cluster-55448.165026	0.55	2.25	8.21	63.36	80.69	196.79	51.13	54.27	72.92
Cluster-55448.167497	6.73	13.12	32.25	165.07	20.16	164.37	0.17	0.12	2.04
Cluster-55448.116476	8.63	8.82	7.35	157.07	23.21	195.84	7.95	30.81	1.27
Cluster-55448.981	0.06	0.05	0	3.16	0.96	5.16	0.21	1.59	0
Cluster-55448.316142	0	0	0	0.17	0	0.17	0	0	0
Cluster-55448.45955	0.22	0.19	0.16	6.59	0.39	25.04	0.45	0	0
Cluster-55448.29667	0	0.27	0.4	3.06	0.28	10.03	0	0	0
Cluster-55448.314871	0	0	0.03	0	0	2.83	0	0	0

Cluster-55448.314872	0	0	0	0	0	0.73	0	0	0
Cluster-49025.0	0	0	0	0	0.07	0.21	0.06	0	0
Cluster-55448.309945	0.25	2.67	14.76	197.78	128.05	1659.49	0.25	0	0
Cluster-55448.311224	0.09	1.07	7.6	200.59	35.3	507.11	0.06	2.09	0
Cluster-55448.312929	0	0	0.03	0.8	1.03	5.03	0	0	0
Cluster-55448.45125	0	0	0	0	0	1.35	0	0	0
Cluster-5386.0	0	0	0	0	0	0.28	0	0	0
Cluster-55448.312568	0	0	0	3.84	1.06	38.95	0	0	0
Cluster-55448.335381	0	0	0	0	0	0.36	0	0	0
Cluster-55448.33567	0	0	0	0	10.68	61.44	0	0	0
Cluster-37324.0	0	0	0	0	0	0.56	0	0	0
Cluster-55448.30798	0	0	0	0	0	10.76	0	0	0
Cluster-55448.227483	0.47	112.93	861.19	2063.81	66.82	3810.71	1.14	0	0
Cluster-55448.16320	0	0	0	0	0	0.54	0	0	0
Cluster-55448.27231	0	0	0	0	0	1.1	0	0	0
Cluster-55448.32305	0	0	0	0	0	11.24	0	0	0
Cluster-55448.24882	0	0	0.79	0.83	0.87	4.01	0	0	0
Cluster-55448.311284	0	0	0	0	0	1.11	0	0	0
Cluster-55448.108285	17.2	21.17	0	51.71	2.46	107.2	0	9.09	0
Cluster-55448.109816	0	32.46	208.96	715.21	89.4	2622.38	0	0	0
Cluster-55448.60253	0	0	0	0	0	0.22	0	0	0
Cluster-55448.232833	0.82	84.03	510.03	823.99	39.08	2017.49	1.9	0	0
Cluster-55448.30021	0	0	0	0	0.15	1.03	0.16	0	0
Cluster-6275.0	0	0	0	0	0	0.45	0	0	0
Cluster-55448.33983	0	141.03	1306.7	1345.42	71.25	4331.32	0	0	0
Cluster-55448.282410	0	0	0	0	0	0.16	0	0	0
Cluster-7139.0	0	0	0	0	0	0.89	0	0	0
Cluster-55448.860	0	0	0	1.05	0	1.87	0	0	0
Cluster-55448.31819	0	2.6	82.8	820.31	195.24	1628.1	0	0	0
Cluster-55448.311609	0	0	0	13.11	0.37	31.92	0	0	0
Cluster-55448.32617	0	2.06	4.17	3.3	0	8	0	0	0
Cluster-44998.0	0	0	0	0	0.17	0.35	0	0	0
Cluster-14428.1	0	0	0	0	0.34	1.55	0	0	0
Cluster-55448.312482	0	0	0	0	0	0.26	0	0	0
Cluster-6093.0	0	0	0.16	0.65	0.32	1.16	0	0	0
Cluster-55448.337756	0	0	0	0	0	0.81	0	0	0
Cluster-59738.0	0	0	0.31	0.16	0	1.63	0	0	0
Cluster-6830.0	0	0	0	0	0.31	0.96	0	0	0
Cluster-55448.27453	0	0.48	2.79	98.1	7.67	91.02	0	0	0
Cluster-55448.234051	0	0	0	0	0	0.15	0	0	0
Cluster-5796.0	0	0	0	0	0	0.52	0	0	0
Cluster-55448.314667	0	0	0	1.69	0.38	2.64	0	0	0
Cluster-55448.33984	0.88	222.3	1468.48	1259.46	131.51	9581.26	1.9	0	0
Cluster-55448.23674	0	0	0	0	0	0.84	0	0	0

Cluster-55448.37225	0	0	0	0.41	0	0.78	0	0	0
Cluster-5800.0	0	0	0.41	0.45	0	0.88	0	0	0
Cluster-55448.310022	0.22	19.69	116.43	387.99	10.28	869.8	0.27	0	0
Cluster-55448.30034	0	0	0.13	0	0.13	1.08	0	0	0
Cluster-55448.312566	0	3.56	5.99	141.13	2.21	171.52	0	0	0
Cluster-55448.297375	0	0	0	1.43	0.62	2.31	0	0	0
Cluster-55448.342758	0	0	0	0	0	2.17	0	0	0
Cluster-55448.312070	0	0	0	0	0	0.77	0	0	0
Cluster-55448.31693	0	0	0	0	0	2.46	0	0	0
Cluster-55448.311426	0	0.91	0.69	0.95	0.93	17.73	0	0	0.13
Cluster-55448.204468	0	0	0	0	0	4.65	0	0	0
Cluster-55448.129164	0	0	0	0	0	0.6	0	0	0
Cluster-55448.30599	0	0	0	0	0	2.44	0	0	0
Cluster-55448.30201	0	0	0	0.87	0	1.45	0	0	0
Cluster-55448.31765	0	0	45.06	75.64	0	134.56	0	0	0
Cluster-5892.0	0	0	0	0.33	0	1.3	0	0	0
Cluster-55448.342229	0	0	0	0	0	0.61	0	0	0
Cluster-55448.153645	0	0	0.41	3.1	1.23	3.88	0	0	0
Cluster-55448.31807	0	0.05	0	49.96	20.68	123.45	0	0	0
Cluster-55448.312515	0	0	0	0	0	0.36	0	0	0
Cluster-6463.0	0	0	0	0	0	0.38	0	0	0
Cluster-14450.0	0	0	0	1.26	0	1.32	0	0	0
Cluster-4036.1	0	0	0.54	1.22	0.55	1.73	0	0	0
Cluster-55448.27984	0	0	0	0.83	0.3	1.48	0	0	0
Cluster-55448.34064	0.46	68.86	577.39	594.34	26.02	1860.55	0.76	0	0

Table S9. Detailed information for all unigenes in the FADs network

Gene_id	Ortholog in Arabidopsis	E-value	Ara_symbol	Annotation in Arabidopsis	PCC-value
Cluster-55448.309693	AT3G12120	1.90E-16	AtFAD2	FAD2|fatty acid desaturase 2	1.0000
Cluster-55448.309945	AT1G23760	3.70E-22	JP630	PG3|POLYGALACTURONASE 3	0.9940
Cluster-5892.0	--	--	--	--	0.9939
Cluster-55448.312567	AT5G01075	8.40E-06	TWS1	Glycosyl hydrolase family 35 protein	0.9917
Cluster-55448.30585	AT4G08290	3.90E-51	UMAMIT20	UMAMIT20|Usually multiple acids move in and out Transporters 20	0.9895
Cluster-55448.314667	--	--	--	--	0.9880
Cluster-55448.47726	AT2G45660	3.30E-19	AGL20	AGL20|AGAMOUS-like 20; ATSOC1|SUPPRESSOR OF OVEREXPRESSION OF CO 1	0.9877
Cluster-55448.250611	AT5G18240	1.10E-15	ATMYR1	ATMYR1|ARABIDOPSIS MYB-RELATED PROTEIN 1; MYR1|myb-related protein 1	0.9872
Cluster-55448.31807	--	--	--	--	0.9860
Cluster-55448.45125	AT1G08170	2.80E-09	--	Histone superfamily protein	0.9857
Cluster-55448.337756	--	--	--	--	0.9857
Cluster-55448.32305	--	--	--	--	0.9857
Cluster-38685.0	AT5G02880	5.60E-10	UPL4	UPL4|ubiquitin-protein ligase 4	0.9857
Cluster-42703.0	AT5G51620	1.20E-09	--	Uncharacterised protein family (UPF0172)	0.9856
Cluster-6463.0	--	--	--	--	0.9855
Cluster-55448.314872	AT1G51720	5.50E-30	--	Amino acid dehydrogenase family protein	0.9854
Cluster-55448.30970	AT2G44470	6.80E-33	BGLU29	BGLU29|beta glucosidase 29	0.9840
Cluster-55448.31693	--	--	--	--	0.9840
Cluster-55448.28465	AT5G23960	1.10E-16	ATTPS21	TPS21|terpene synthase 21	0.9835
Cluster-55448.29392	AT5G58080	2.70E-07	ARR18	ARR18|response regulator 18	0.9833
Cluster-55448.30599	--	--	--	--	0.9830
Cluster-55448.311247	AT3G29152	3.00E-08	--	Bifunctional inhibitor/lipid-transfer protein/seed storage 2S albumin superfamily protein	0.9828
Cluster-55448.143429	AT5G11170	2.20E-68	UAP56a	UAP56a|homolog of human UAP56 a	0.9826
Cluster-55448.311224	AT1G23760	6.30E-21	JP630	PG3|POLYGALACTURONASE 3	0.9823
Cluster-32876.0	AT5G13410	2.20E-48	--	FKBP-like peptidyl-prolyl cis-trans isomerase family protein	0.9819
Cluster-55448.297375	--	--	--	--	0.9809
Cluster-55448.32905	AT4G30950	7.90E-28	FAD6	FAD6|fatty acid desaturase 6; FADC|FATTY ACID DESATURASE C; SFD4|STEAROYL DESATURASE DEFICIENCY 4	0.9807

Cluster-7139.0	--	--	--	--	0.9795
Cluster-55448.312070	--	--	--	--	0.9793
Cluster-55448.210681	AT4G04970	6.30E-08	ATGSL01	ATGSL01|GLUCAN SYNTHASE LIKE 1; ATGSL1|GLUCAN SYNTHASE LIKE-1; GSL1|glucan synthase-like 1	0.9791
Cluster-55448.31819	--	--	--	--	0.9789
Cluster-55448.311426	--	--	--	--	0.9788
Cluster-4036.1	--	--	--	--	0.9772
Cluster-55448.51735	AT3G48530	4.70E-10	KINγ	KING1|SNF1-related protein kinase regulatory subunit gamma 1	0.9764
Cluster-55448.30034	--	--	--	--	0.9758
Cluster-55448.204468	--	--	--	--	0.9755
Cluster-55448.16320	--	--	--	--	0.9749
Cluster-55448.30798	--	--	--	--	0.9748
Cluster-55448.342229	--	--	--	--	0.9746
Cluster-37324.0	--	--	--	--	0.9744
Cluster-5800.0	--	--	--	--	0.9740
Cluster-55448.31765	--	--	--	--	0.9728
Cluster-55448.312515	--	--	--	--	0.9724
Cluster-55448.227483	--	--	--	--	0.9723
Cluster-55448.335381	--	--	--	--	0.9722
Cluster-14450.0	--	--	--	--	0.9705
Cluster-55448.167497	AT2G02130	1.40E-06	LCR68	LCR68|low-molecular-weight cysteine-rich 68	0.9700
Cluster-55448.312929	AT1G12740	2.40E-48	CYP87A2	CYP87A2|cytochrome P450, family 87, subfamily A, polypeptide 2	0.9692
Cluster-55448.30021	--	--	--	--	0.9691
Cluster-14428.1	--	--	--	--	0.9668
Cluster-55448.108285	--	--	--	--	0.9662
Cluster-55448.33567	--	--	--	--	0.9661
Cluster-44998.0	--	--	--	--	0.9648
Cluster-55448.31704	AT4G01970	1.10E-15	AtSTS	AtSTS|stachyose synthase; RS4|raffinose synthase 4	0.9641
Cluster-49025.0	AT1G45170	1.30E-15	--	unknown protein	0.9640
Cluster-55448.116476	AT1G78560	2.70E-41	--	Sodium Bile acid symporter family	0.9621
Cluster-55448.153645	--	--	--	--	0.9612
Cluster-55448.316142	AT1G68840	5.50E-07	AtRAV2	EDF2|ETHYLENE RESPONSE DNA BINDING FACTOR 2; RAP2.8|RELATED TO AP2 8; RAV2|related to ABI3/VP1 2; TEM2|TEMPRANILLO 2	0.9607
Cluster-55448.232833	--	--	--	--	0.9604
Cluster-55448.312566	--	--	--	--	0.9602
Cluster-55448.32617	--	--	--	--	0.9597
Cluster-55448.27453	--	--	--	--	0.9580
Cluster-55448.27984	--	--	--	--	0.9546
Cluster-6830.0	--	--	--	--	0.9528
Cluster-55448.23674	--	--	--	--	0.9473
Cluster-55448.311609	--	--	--	--	0.9465

Cluster-54354.0	AT3G57310	6.70E-07	--	Bifunctional inhibitor/lipid-transfer protein/seed storage 2S albumin superfamily protein	0.9450
Cluster-55448.309691	AT4G30950	1.80E-25	FAD6	FAD6|fatty acid desaturase 6; FADC|FATTY ACID DESATURASE C; SFD4|STEAROYL DESATURASE DEFICIENCY 4	0.9444
Cluster-55448.205982	AT2G35460	2.00E-06	--	Late embryogenesis abundant (LEA) hydroxyproline-rich glycoprotein family	0.9200
Cluster-55448.981	AT1G73965	3.10E-06	CLE13	CLE13|CLAVATA3/ESR-RELATED 13	0.9153
Cluster-55448.309691	AT4G30950	1.80E-25	FAD6	FAD6|fatty acid desaturase 6; FADC|FATTY ACID DESATURASE C; SFD4|STEAROYL DESATURASE DEFICIENCY 4	1.0000
Cluster-55448.981	AT1G73965	3.10E-06	CLE13	CLE13|CLAVATA3/ESR-RELATED 13	0.9478
Cluster-55448.309693	AT3G12120	1.90E-16	AtFAD2	FAD2|fatty acid desaturase 2	0.9444
Cluster-55448.30034	--	--	--	--	0.9331
Cluster-55448.33567	--	--	--	--	0.9309
Cluster-55448.32905	AT4G30950	7.90E-28	FAD6	FAD6|fatty acid desaturase 6; FADC|FATTY ACID DESATURASE C; SFD4|STEAROYL DESATURASE DEFICIENCY 4	0.9306
Cluster-6830.0	--	--	--	--	0.9301
Cluster-55448.23674	--	--	--	--	0.9296
Cluster-44998.0	--	--	--	--	0.9287
Cluster-55448.31807	--	--	--	--	0.9265
Cluster-55448.309945	AT1G23760	3.70E-22	JP630	PG3|POLYGALACTURONASE 3	0.9255
Cluster-55448.27984	--	--	--	--	0.9230
Cluster-55448.312929	AT1G12740	2.40E-48	CYP87A2	CYP87A2|cytochrome P450, family 87, subfamily A, polypeptide 2	0.9200
Cluster-14428.1	--	--	--	--	0.9199
Cluster-55448.116476	AT1G78560	2.70E-41	--	Sodium Bile acid symporter family	0.9163
Cluster-55448.153645	--	--	--	--	0.9149
Cluster-55448.129164	--	--	--	--	0.9001
Cluster-55448.312482	--	--	--	--	0.8554
Cluster-55448.165026	AT2G02860	1.90E-73	ATSUC3	ATSUC3|SUCROSE TRANSPORTER 3; SUT2|sucrose transporter 2	0.8468
Cluster-5796.0	--	--	--	--	0.8435
Cluster-55448.230922	AT3G48190	2.60E-09	ATATM	ATATM|ATAXIA-TELANGIECTASIA MUTATED; PIG1|pcd in male gametogenesis 1	0.8281
Cluster-55448.118765	AT5G09320	7.70E-10	VPS9B	VPS9B|Vacuolar sorting protein 9 (VPS9) domain	0.7820
Cluster-55448.32905	AT4G30950	7.90E-28	FAD6	FAD6|fatty acid desaturase 6; FADC|FATTY ACID DESATURASE C; SFD4|STEAROYL DESATURASE DEFICIENCY 4	1.0000
Cluster-55448.30970	AT2G44470	6.80E-33	BGLU29	BGLU29|beta glucosidase 29	0.9939

Cluster-55448.31807	--	--	--	--	0.9930
Cluster-55448.109816	--	--	--	--	0.9873
Cluster-55448.312568	--	--	--	--	0.9857
Cluster-55448.314388	AT2G45660	3.60E-19	AGL20	AGL20|AGAMOUS-like 20; ATSOC1|SUPPRESSOR OF OVEREXPRESSION OF CO 1	0.9851
Cluster-55448.250611	AT5G18240	1.10E-15	ATMYR1	ATMYR1|ARABIDOPSIS MYB-RELATED PROTEIN 1; MYR1|myb-related protein 1	0.9849
Cluster-55448.310022	--	--	--	--	0.9840
Cluster-55448.33984	--	--	--	--	0.9829
Cluster-55448.232833	--	--	--	--	0.9809
Cluster-55448.309693	AT3G12120	1.90E-16	AtFAD2	FAD2|fatty acid desaturase 2	0.9807
Cluster-55448.309945	AT1G23760	3.70E-22	JP630	PG3|POLYGALACTURONASE 3	0.9804
Cluster-55448.29667	AT1G68600	3.70E-09	--	Aluminium activated malate transporter family protein	0.9793
Cluster-55448.342758	--	--	--	--	0.9790
Cluster-55448.311284	--	--	--	--	0.9785
Cluster-55448.227483	--	--	--	--	0.9785
Cluster-55448.27231	--	--	--	--	0.9781
Cluster-55448.314871	AT1G51720	1.20E-33	--	Amino acid dehydrogenase family protein	0.9779
Cluster-55448.30201	--	--	--	--	0.9775
Cluster-55448.51735	AT3G48530	4.70E-10	KINγ	KING1|SNF1-related protein kinase regulatory subunit gamma 1	0.9771
Cluster-55448.312929	AT1G12740	2.40E-48	CYP87A2	CYP87A2|cytochrome P450, family 87, subfamily A, polypeptide 2	0.9768
Cluster-55448.30585	AT4G08290	3.90E-51	UMAMIT20	UMAMIT20|Usually multiple acids move in and out Transporters 20	0.9763
Cluster-55448.89390	AT2G05530	6.10E-08	--	Glycine-rich protein family	0.9761
Cluster-55448.210681	AT4G04970	6.30E-08	ATGSL01	ATGSL01|GLUCAN SYNTHASE LIKE 1; ATGSL1|GLUCAN SYNTHASE LIKE-1; GSL1|glucan synthase-like 1	0.9757
Cluster-55448.108285	--	--	--	--	0.9756
Cluster-7139.0	--	--	--	--	0.9754
Cluster-55448.31693	--	--	--	--	0.9751
Cluster-55448.282410	--	--	--	--	0.9749
Cluster-55448.70214	AT5G11380	1.10E-19	DXPS3	DXPS3|1-deoxy-D-xylulose 5-phosphate synthase 3; DXS3|1-deoxy-D-xylulose 5-phosphate (DXP) synthase 3	0.9749
Cluster-55448.252811	AT3G56310	8.60E-06	--	Melibiase family protein	0.9749
Cluster-55448.34064	--	--	--	--	0.9745
Cluster-5386.0	--	--	--	--	0.9742
Cluster-55448.33983	--	--	--	--	0.9738
Cluster-55448.60253	--	--	--	--	0.9735
Cluster-6275.0	--	--	--	--	0.9734
Cluster-55448.76989	AT3G08490	2.90E-	--	--	0.9733

		08			
Cluster-55448.30599	--	--	--	--	0.9717
Cluster-55448.45955	AT1G68600	4.30E-47	--	Aluminium activated malate transporter family protein	0.9685
Cluster-55448.860	--	--	--	--	0.9664
Cluster-55448.27984	--	--	--	--	0.9663
Cluster-55448.309421	AT2G05530	4.10E-08	--	Glycine-rich protein family	0.9657
Cluster-55448.37225	--	--	--	--	0.9656
Cluster-55448.312566	--	--	--	--	0.9636
Cluster-55448.297375	--	--	--	--	0.9631
Cluster-55448.116476	AT1G78560	2.70E-41	--	Sodium Bile acid symporter family	0.9620
Cluster-55448.24882	--	--	--	--	0.9608
Cluster-6093.0	--	--	--	--	0.9602
Cluster-55448.31704	AT4G01970	1.10E-15	AtSTS	AtSTS|stachyose synthase; RS4|raffinose synthase 4	0.9577
Cluster-54354.0	AT3G57310	6.70E-07	--	Bifunctional inhibitor/lipid-transfer protein/seed storage 2S albumin superfamily protein	0.9437
Cluster-59738.0	--	--	--	--	0.9346
Cluster-55448.981	AT1G73965	3.10E-06	CLE13	CLE13|CLAVATA3/ESR-RELATED 13	0.9306
Cluster-55448.309691	AT4G30950	1.80E-25	FAD6	FAD6|fatty acid desaturase 6; FADC|FATTY ACID DESATURASE C; SFD4|STEAROYL DESATURASE DEFICIENCY 4	0.9306
Cluster-55448.205982	AT2G35460	2.00E-06	--	Late embryogenesis abundant (LEA) hydroxyproline-rich glycoprotein family	0.9215
Cluster-55448.99355	AT4G21720	5.60E-08	--	unknown protein	0.8904
Cluster-55448.232891	AT2G42190	1.20E-21	--	unknown protein	0.8725
Cluster-55448.312482	--	--	--	--	0.8508
Cluster-55448.234051	--	--	--	--	0.7846
